# *Acinetobacter baumannii* Repeatedly Evolves a Hypermutator Phenotype in Response to Tigecycline That Effectively Surveys Evolutionary Trajectories to Resistance

**DOI:** 10.1371/journal.pone.0140489

**Published:** 2015-10-21

**Authors:** Troy G. Hammerstrom, Kathryn Beabout, Thomas P. Clements, Gerda Saxer, Yousif Shamoo

**Affiliations:** Department of Biosciences, Rice University, Houston, Texas, United States of America; Ella Foundation, INDIA

## Abstract

The evolution of hypermutators in response to antibiotic treatment in both clinical and laboratory settings provides a unique context for the study of adaptive evolution. With increased mutation rates, the number of hitchhiker mutations within an evolving hypermutator population is remarkably high and presents substantial challenges in determining which mutations are adaptive. Intriguingly however, hypermutators also provide an opportunity to explore deeply the accessible evolutionary trajectories that lead to increased organism fitness, in this case the evolution of antibiotic resistance to the clinically relevant antibiotic tigecycline by the hospital pathogen *Acinetobacter baumannii*. Using a continuous culture system, AB210M, a clinically derived strain of *A*. *baumannii*, was evolved to tigecycline resistance. Analysis of the adapted populations showed that nearly all the successful lineages became hypermutators via movement of a mobile element to inactivate *mutS*. In addition, metagenomic analysis of population samples revealed another 896 mutations that occurred at a frequency greater than 5% in the population, while 38 phenotypically distinct individual colonies harbored a total of 1712 mutations. These mutations were scattered throughout the genome and affected ~40% of the coding sequences. The most highly mutated gene was *adeS*, a known tigecycline-resistance gene; however, *adeS* was not solely responsible for the high level of TGC resistance. Sixteen other genes stood out as potentially relevant to increased resistance. The five most prominent candidate genes (*adeS*, *rpsJ*, *rrf*, *msbA*, and *gna*) consistently re-emerged in subsequent replicate population studies suggesting they are likely to play a role in adaptation to tigecycline. Interestingly, the repeated evolution of a hypermutator phenotype in response to antibiotic stress illustrates not only a highly adaptive strategy to resistance, but also a remarkably efficient survey of successful evolutionary trajectories.

## Introduction

Adaptation to antibiotics, like adaptation to any environment, can occur through diverse evolutionary processes. Essentially, any genetic mechanism that improves the fitness of an organism undergoing antibiotic selection can be observed in the clinical record. Single nucleotide polymorphisms (SNPs), large and small insertions and deletions, movement of mobile genetic elements, and horizontal gene transfer all play important roles in the evolution of multi-drug resistant pathogens [1]. One frequent outcome of selection in both patients undergoing antibiotic therapies and *in vitro* laboratory evolution is the generation of hypermutators. These hypermutators often have 10–100 fold higher mutation rates than their ancestors and can provide a significant adaptive advantage over slower evolving strains. Hypermutators in clinical settings have been observed in many species including *Pseudomonas aeruginosa*, *Escherichia coli*, *Salmonella enterica*, and *Neisseria meningitidis* (reviewed in [1]). While hypermutator strains confer a significant adaptive advantage in the short run, they also burden the organism with a plethora of mutations most of which are non-adaptive and may reduce overall fitness to a range of environmental conditions [2–5]. In bacteremia or other infections of niche environments within a patient, the short-term adaptive benefits of a hypermutator phenotype can prove very advantageous as the role of purifying selection is diminished under such strong selection for a single adaptive phenotype (e.g. resistance).

In this report, we studied the evolution of a hypermutator strain of *Acinetobacter baumannii* AB210 during antibiotic selection to the frontline antibiotic tigecycline (TGC). As a hospital-acquired pathogen, *A*. *baumannii* causes 12,000 infections per year in the United States in critically ill patients [6]. Strains of *A*. *baumannii* have rapidly acquired antibiotic resistance via upregulation of efflux pumps and horizontal gene transfer which have severely decreased the efficacy of most antibiotics including TGC [7]. TGC is in the tetracycline family but the first of the glycylcycline class of antibiotics. The molecule binds reversibly to the A site of the ribosome and inhibits translation [8]. TGC-susceptible *A*. *baumannii* AB210 was isolated originally from an intra-abdominal infection. After one week of TGC therapy, AB211 was isolated and identified as a TGC-resistant, hypermutator strain. A large deletion in AB211 truncated *mutS*, which encodes an essential protein of the DNA mismatch repair pathway (reviewed in [9]) leading to a hypermutator phenotype [10].

One prominent mechanism for TGC resistance is overexpression of the resistance-nodule-diffusion family of efflux pumps (RND pumps). There are three RND pumps in *A*. *baumannii*, AdeABC, AdeFGH, and AdeIJK, which are controlled by the transcriptional regulators AdeRS, AdeL, and AdeN, respectively. Mutations in AdeRS, AdeL, and AdeN lead to increased expression of the RND pumps and cause decreased susceptibility to several classes of antibiotics [11–16]. For example, comparative genomic sequencing of AB210 and AB211 revealed a mutation in *adeS* that increased expression of AdeABC thus conferring TGC resistance [17,18].

To identify genes associated with TGC resistance as well as their relative importance, we cultured *A*. *baumannii* AB210M, a derivative of AB210, to achieve high levels of TGC resistance. We used a selection and cultivation scheme that favors the formation of strongly polymorphic populations and biofilms in a novel bioreactor configuration under well-controlled parameters such as drug concentration, metabolic respiration rate, and the maintenance of non-limiting nutrient concentrations. Using our bioreactor system, we were able to recapitulate the evolution of the hypermutator phenotype in two separate populations of AB210M and then deconstruct the tremendous number of evolutionary trajectories leading to high levels of TGC resistance. We observed thousands of mutations of varying types and classes and then identified a subset of those most likely to be involved with resistance. By surveying samples of the adapting population on each day of the trial, we were able to determine the order and frequency of adaptive alleles and thus gain insights into the relative importance of each. One interesting aspect of the evolution of a hypermutator population is the extent to which they saturate the genome with mutations and provide a rich and comprehensive exploration of the available evolutionary trajectories leading to a new phenotype, in this case TGC resistance. While it is challenging to analyze and comprehensively validate this wealth of mutational data, we are able to identify clinically relevant pathways as well as discover several new pathways that may contribute to emerging TGC resistance in *A*. *baumannii* and potentially other Gram-negative pathogens like *Klebsiella pneumoniae* and *E*. *coli*.

## Results

### Adaptation of a clinical isolate of *A*. *baumannii* to increasing but sub-inhibitory concentrations of tigecycline leads to a rapid rise to resistance

*A*. *baumannii* strain AB210M gradually evolved resistance to tigecycline (TGC) over 26 days in a continuous culture in two independent experiments (Trial 1 and Trial 2). The final populations were cultured in 16 μg/ml TGC, which was twice the minimal inhibitory concentration (MIC) of AB211, the clinical TGC-resistant isolate, and greater than the clinical breakpoint (>8 μg/ml TGC) [17]. Each culture contained ~10^10^ cfu with ~36 generations per day. We noted that biofilm communities were readily visible on the stainless steel and borosilicate glass surfaces within the bioreactor as early as 72 hr after inoculation.

We took two approaches to examine the evolving hypermutator populations. In the first approach, we isolated total DNA from the planktonic population, metal and glass biofilms at the end of the trials and performed a metagenomic analysis to identify all the alleles that rose above a frequency of 5% (Table 1 Metagenomic Endpoint Populations). In the second approach, we characterized 90 isolates from each trial to capture genetically distinct, but perhaps less common, evolutionary trajectories that might not be revealed as readily by the metagenomic analysis. The colonies originated from the biofilms on the glass and metal surfaces as well as the planktonic community. We characterized the isolates with a series of phenotypic tests including MIC, growth rate, and swarming motility and selected a set of clones for whole genome sequencing (Table 1 TGC^R^ Endpoint Isolates and S1 Text). This second approach also allowed us to identify genetic linkages suggested from the metagenomic analysis.

**Table 1:** Description of samples sequenced from two independent adaptation trials.

Sample	Trial 1	Trial 2*
Metagenomic Endpoint Populations	3	3
TGC^R^ Endpoint Isolates	10	28
Daily Metagenomic Populations	26	-

*Daily metagenomic populations were not sequenced from Trial 2.

### Deletions in the *mutS* locus evolve repeatedly and lead to a hypermutator phenotype

**Fig 1:**
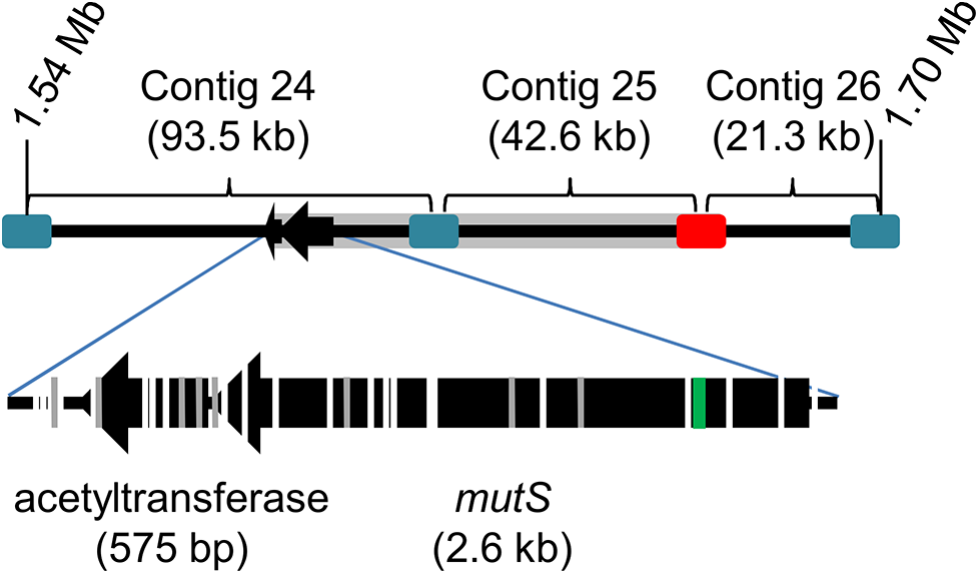
~60 kb deletions begin near and extend into *mutS* to confer the hypermutator phenotype. The top line demonstrates contigs 24–26 separated by mobile elements IS *Aba-1* (blue) and IS15 D1 (red) and the deleted region (gray). The genomic position of the region can be compared to Figs 2C and 4C. In the lower portion of the figure, the *mutS* gene is enlarged to identify IS15 D1 insertion sites. White breaks in *mutS* and the adjacent gene indicate positions where IS15 D1 inserted in one or more polymorphic samples. Gray lines mark where IS15 D1 inserted in the clonal isolates. The deletion in clinical isolate AB211 is indicated (green).

Hypermutator strains arose rapidly (>50% of the population on Day 5 in Trial 1, S2 Fig) and comprised all but two of the successful evolutionary trajectories. The hypermutator phenotypes were due to ~60kb deletions that began within or included *mutS* and the last ~14kb of contig AEOXM024 and all of contig AEOXM025 (contig names are abbreviated to the last two digits, Fig 1) [10]. The frequency of the deletion nears fixation (>95%) by Day 10 of Trial 1 and remains at that level through the end of the adaptation (S2 Fig). Within individual clones, the exact size of the deletion varied from 56–69 kb and included approximately 49 genes depending on the specific event. The movement of an insertion element (IS15 D1, [19]) produced this family of *mutS*-associated deletions. Incorporating all the sequencing results from endpoint stains and metagenomic analysis of population samples from both trials, we identified 26 different IS15 D1 insertion sites in the *mutS* region. We found examples where the IS15 D1 element inserted in either orientation into the *mutS* locus to delete *mutS*. Interestingly, a similar deletion was found in the clinical resistant isolate AB211 and produced a hypermutator phenotype in the patient [18]. However, in the case of AB211, the deletion of *mutS* was not made by IS15 D1 but by an unknown mechanism. The variety and consistency of events leading to the hypermutator phenotype suggests a selective benefit for hypermutators both in clinical and experimental conditions.

### Analysis of polymorphic endpoint populations shows significant parallel adaptation

**Fig 2:**
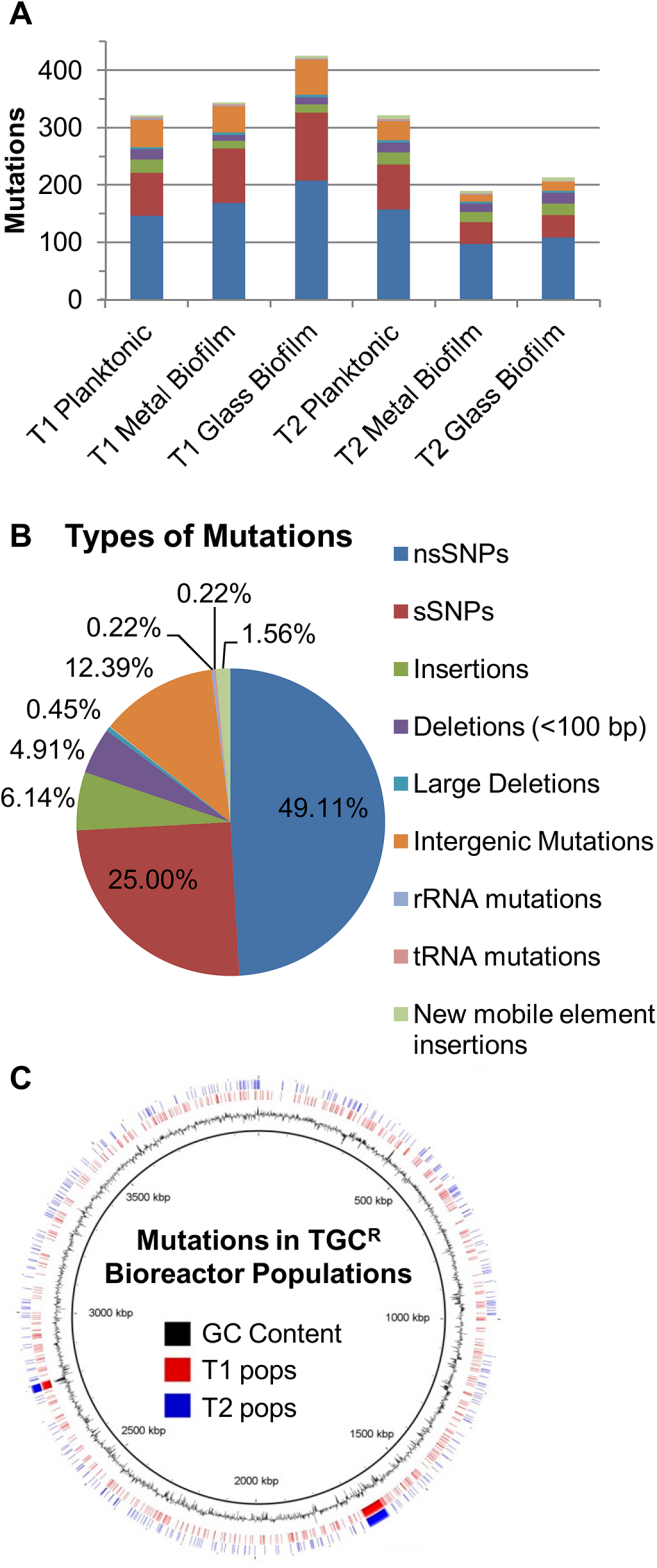
Metagenomic analysis of endpoint populations illustrated the diversity of mutations in the adapting populations. Mutations with a frequency ≥5% isolated from different locations and trials of the bioreactor are shown: (A) the number of mutations for each spatial sample; and (B) total frequency and types of mutations across all populations. (C) Lines indicate locations of the mutations in the AB210M genome (Trial 1, red; Trial 2, blue).

Based on our previous experience with adaptation of populations to sub-inhibitory concentrations of antibiotics and weak to moderate selection conditions with model organisms, we expected to observe polymorphic populations with many potential evolutionary trajectories leading to adaption to TGC [20–23]. For the first experiment (Trial 1), sequencing data from genomic DNA isolated from the planktonic, metal and glass biofilms contained 570 unique mutations, which was substantially greater than our previous experiments. The overall number of mutations from each sample location was comparable suggesting that hypermutators occupied all the sampled spatial niches (Fig 2A). Additionally, we observed different mutations or different frequencies of the same mutation in each sampling site suggesting that each spatial sample may have evolved independently (S1 Fig). Strains deficient in *mutS* have a strong bias to produce transition mutations [24,25], which was also detected in our samples as 485 of the 489 (99.2%) SNPs in coding regions were transition mutations. *In toto*, 422 of 3741 annotated genes were mutated in the populations with the mutations spread throughout the genome (Fig 2C). In the second adaptation experiment (Trial 2), hypermutators again arose within the adapting population generating comparable numbers and frequencies of mutational events (Fig 2A and 2B and S1 Table). Combining the data from endpoint populations from Trial 1 and 2 (Table 1 and S1 Table), we identified 440 non-synonymous SNPs (nsSNPs, 49.11%), 224 synonymous SNPs (sSNPs, 25.00%), 55 insertions (6.14%), 44 deletions <100 bp (4.91%), 4 large deletions (>100 bp, 0.45%), 111 intergenic mutations (12.39%), 14 new junctions due to mobilization of insertion elements (1.56%), 2 SNPs in a ribosomal gene (0.22%), and 2 SNPs in transfer RNA genes (0.22%) (Fig 2B, more details of the large deletions are included in the S1 Text and S2 Fig). Like Trial 1, mutations were distributed throughout the genome (Fig 2C).

**Fig 3:**
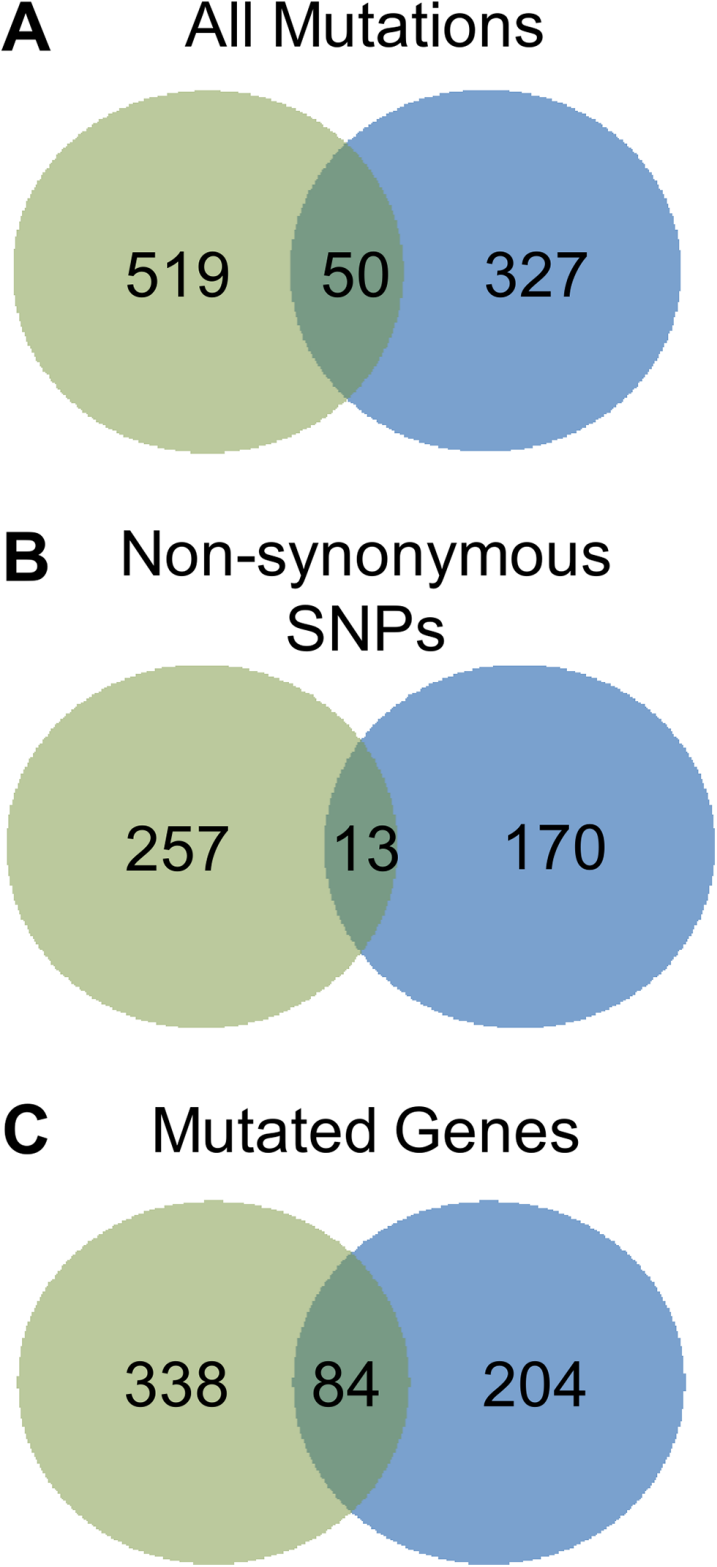
Parallel evolution of endpoint populations was observed from Trials 1 and 2. Venn diagrams represent the number of (A) all mutations, (B) nsSNPs, or (C) genes that were observed in the individual trials (Trial 1, green; Trial 2, blue).

Given the large number of mutations and the likelihood that the vast majority will be non-adaptive (hitchhiker mutations), we initially focused on those mutations that were observed in both trials suggesting parallel adaptive evolution of specific alleles [22]. As shown in Fig 3, there were a total of 896 unique mutations in the two populations; however, only 50 were identical in both trials: 13 nsSNPs, 6 sSNPs, 12 insertions, 8 deletions, 4 large deletions, 4 intergenic mutations, 1 ribosomal gene SNP, and 2 tRNA gene SNPs. When examining the mutations at the level of the gene, 626 genes were mutated in at least one trial, but only 84 genes were mutated in both (Fig 3C). Genes with multiple mutations within their coding region had an average of 2.5 mutations; however 12 genes had four or more mutations, suggesting very strong and non-random selection for those genes. To determine the likelihood that a particular gene contained the identified number of mutations at random, we used Fisher’s Exact Test to compare the number of unique mutations throughout the genome to the number of mutations in a particular gene. Using the sequential Bonferroni correction for multiple testing, we determined that *p* values < 8.02 x 10^−5^ were statistically significant. Our second test to identify adaptive mutations was the presence of identical nsSNPs across Trials 1 and 2. For example *adeS* ^A94V^, *adeS* ^A130V^, and *adeS* ^T153A^ appeared in both trials and *adeS* had 11 unique mutations in the endpoint population samples from both trials (*p* <0.001, Table 2 and S1 Table). Indeed, mutations to *adeS* have been identified previously in clinical studies as providing a substantial increase in TGC resistance [18,26–28] and were strongly selected for as seen in Table 2.

**Table 2:** Candidate TGC resistance genes.

					Samples with mutations		Fisher’s Exact Test (*p* value)	
GenBank ID	Gene	Description	Total Muts	Identical nsSNPs	Mixed Pops	Clones	Mixed Pops	Clones
Transcription and Translation Machinery								
WM39_01000	*rnpA*	Ribonuclease P protein component	3	0	3	9	0.003	<0.001
WM39_02610	tRNA^Met^	tRNA‑Met‑CAT	1	1	6	18	0.016	0.031
WM39_02985	*rppH*	Adenosine (5')‑pentaphospho‑ (5'')‑adenosine pyrophosphohydrolase	9	0	5	5	<0.001	0.001
WM39_12295	*rrf*	Ribosome recycling factor	9	1	6	17	<0.001*	<0.001*
WM39_15945	*rpoD*	RNA polymerase sigma factor D	8	0	1	5	0.001	<0.001*
WM39_17565	*rpsJ*	SSU ribosomal protein S10	3	2	6	18	0.002	0.008
**Outer membrane permeability**								
WM39_01255	*wzc*	Tyrosine-protein kinase	7	1	4	8	0.002	0.016
WM39_01270	*gna*	UDP-N-acetyl-glucos-amine dehydrogenase	9	0	6	22	<0.001*	<0.001
WM39_16595	*ompA*	Outer membrane protein A precursor	2	1	4	5	0.215	0.369
**Efflux pumps and their regulators**								
WM39_02895	*abeM*	MATE family transporter	4	1	5	19	0.038	0.021
WM39_09850	*msbA*	Lipid A export ATP‑binding/ permease protein	14	2	4	24	<0.001*	<0.001*
WM39_10975	*adeS*	Two-component histidine kinase of *adeABC*	16	4	6	38	<0.001*	<0.001*
**Unknown Role**								
WM39_05440		hypothetical protein	3	0	3	10	<0.001	0.002
WM39_06375	*ureC*	Urease alpha subunit	4	1	6	15	0.057	0.167
WM39_11825	*pcaF*	Acetyl‑CoA acetyl-transferase @ Beta‑ketoadipyl CoA thiolase	9	0	4	2	<0.001*	<0.001*
WM39_12540		hypothetical protein	12	0	1	6	0.421	<0.001*
WM39_12950		RNA‑binding protein Hfq / domain of unknown function	4	1	2	6	0.108	0.196

* The *p* values were significant after applying the sequential Bonferroni correction for multiple tests.

### Endpoint strains provide evolutionary trajectories and genetic linkages to TGC resistance

The evolution of hypermutators throughout the populations led to a very large number of hitchhiking mutations that made identification of rare adaptive mutations and elucidation of the successful adaptive trajectories more challenging. To gain insight into the genetic diversity and linkages responsible for TGC resistance, we selected 90 colonies from the endpoint populations of each trial. Using phenotypic assays, we differentiated the isolates using clustering analyses and observed eight phenotypic clusters for each trial (see S1 Text and S3 Fig). Notably, most isolates had a TGC MIC greater than or equal to 128 μg/ml and all isolates had TGC MIC values above 16 μg/ml. In addition, the colonies displayed a wide range of fitness defects as observed via their replication rate in non-selective medium (S4 Fig). After discovering the hypermutator phenotype by sequencing 10 Trial 1 isolates, we sequenced 28 isolates from Trial 2 to widen the analysis of the endpoint lineages (Table 1, S4 Fig). We selected colonies for sequencing from the different phenotypic clusters in a biased fashion to identify rare phenotypes and to complement our more general understanding of adaptation that resulted from the allelic frequencies readily obtained from our population metagenomic study.

**Fig 4:**
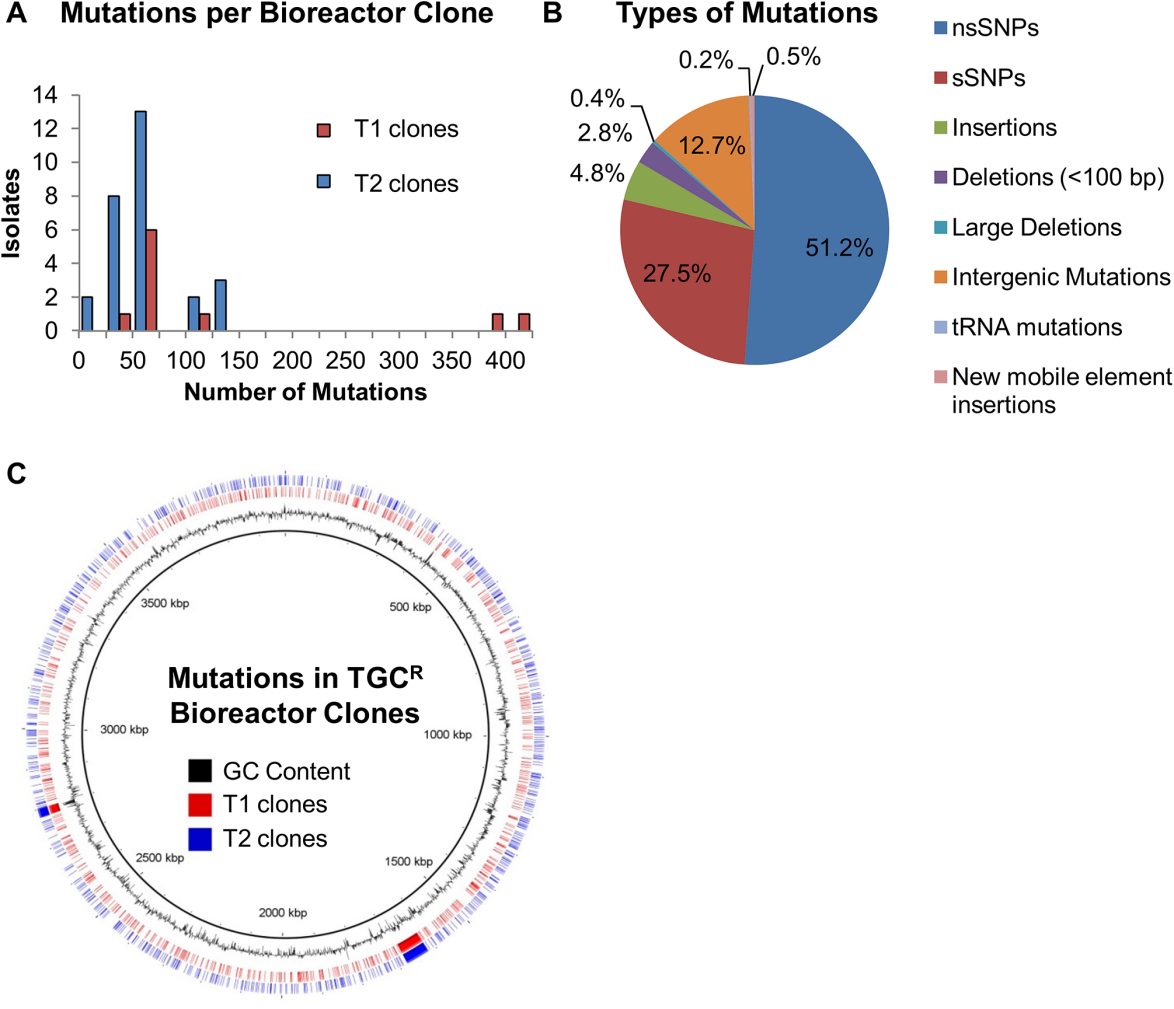
Whole genome sequencing of endpoint clonal isolates illustrated a wide range of mutations. (A) Total number of mutations in each clone. The two non-hypermutators (T2_F9 and T2_H3) appear on the far left of the plot with <10 mutations while T1_H11 and T1_B5 each had 395 or 415 mutations. (B) Types of mutations for the collection of 38 isolates. (C) Locations of the mutations in the AB210M genome (Trials 1, red; Trial 2, blue).

The ten isolates chosen from Trial 1 contained an average of 82 mutations (range = 50–415, Fig 4A). Two clones (T1_B5 and T1_H11) contained the most mutations with 415 and 395 each. Altogether, there were 821 unique mutations of various types (Fig 4B) in 577 genes distributed throughout the genome (Fig 4C). The strains from Trial 2 contained 918 unique mutations and an average of 32 mutations per strain (range = 3–136). Four strains (T2_A8, T2_C8, T2_D1, and T2_D5) had the most mutations ranging from 118 to 136 (Fig 4A). Surprisingly, two strains (T2_F9 and T2_H3) only contained six and three mutations each and were not hypermutators; however, they were resistant to 128 μg/ml TGC similar to the hypermutator isolates (S4 Fig). These were the only isolates that contained an intact *mutS* locus. Like the Trial 1 results, there were many different types of mutations spread throughout the genome (Fig 4B and 4C). In all, the Trial 2 clones had mutations in 667 genes.

**Fig 5:**
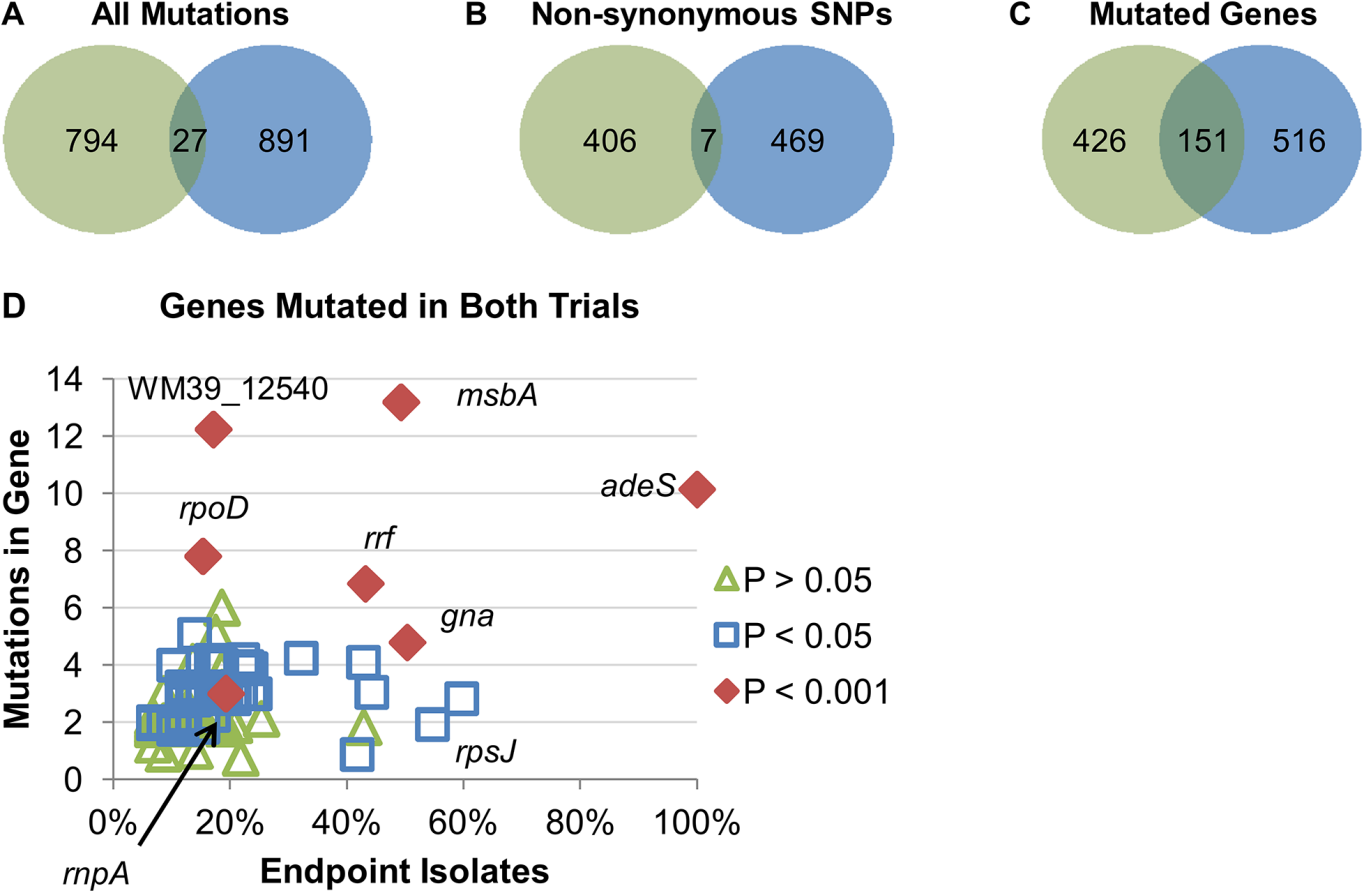
Parallel evolution suggests genes that may contribute to TGC resistance. Venn diagrams represent (A) the number of mutations, (B) nsSNPs, or (C) genes that were observed in the individual trials or combined (Trial 1, green; Trial 2 blue). (D) Genes mutated in both trials were plotted based on the number of mutations in the gene and the percentage of clones that contained at least one mutation in the gene. False noise was added to the number of mutations in each gene to separate the spots. *P* values were calculated using Fisher’s Exact Test. Genes with *p* values < 0.001 are labeled. Due to its role in TGC resistance, *rpsJ* was also labeled.

Overall, the mutations in the endpoint isolates affected 1093 genes, over 25% of the genes in AB210M. While a large number of genes were mutated, only 145 genes were mutated during both trials (Fig 5A–5C). A majority of the genes in common had two mutations, but several had three or more. For example, *msbA* contained 13 mutations (all nsSNPs), a hypothetical protein (WM39_12540) had 12 mutations, and *adeS* contained 10 mutations. Of these genes, only *adeS* has a confirmed role in TGC resistance. We reasoned that within the 145 genes mutated in both trials, genes with multiple mutations in many different isolates were more likely to be adaptive. Using the Fisher Exact Test, 51 genes had *p* values <0.05, and 7 genes had *p* values <0.001 (Table 2). We overlaid the statistical test results on a scatter plot of the number of mutations per gene by the percentage of clones (weighted by trial) having at least one mutation in that gene (Fig 5D). Six of the seven genes with *p* values <0.001 were in the upper right portion of the plot consistent with being an adaptive allele. When the sequential Bonferroni correction was applied to the *p* values, *adeS*, *msbA*, and *rrf* were statistically significant. In excellent agreement with the metagenomic endpoint population analysis, *adeS* was the most prominent gene as all the clones harbored mutations in this gene. Most of the genes in the plot clustered on the bottom left suggesting that these genes are less likely to play a role in TGC resistance consistent with being hitchhikers.

### Mutations to *adeS* alone do not confer the high levels of TGC resistance

We identified mutations in *adeS* in every endpoint clone and population from both bioreactor trials (Table 2). It has been shown in multiple clinical strains and *in vitro* isolates that mutations in *adeS* decrease TGC susceptibility of *A*. *baumannii*. The TGC MIC values of our strains were similar to clinical strains described by Navon-Venezia and colleagues (128 μg/ml TGC) [29] but higher than that observed for the TGC-resistant clinical isolate AB211 (8 μg/ml TGC). Hornsey and colleagues demonstrated that TGC resistance in AB211 was due to overexpression of the *adeABC* efflux pump via the *adeS* ^A94V^ mutation [17]. Since the TGC MICs of our adapted strains were greater than AB211, we postulated that additional mutations were needed to confer the MICs observed.

**Fig 6:**
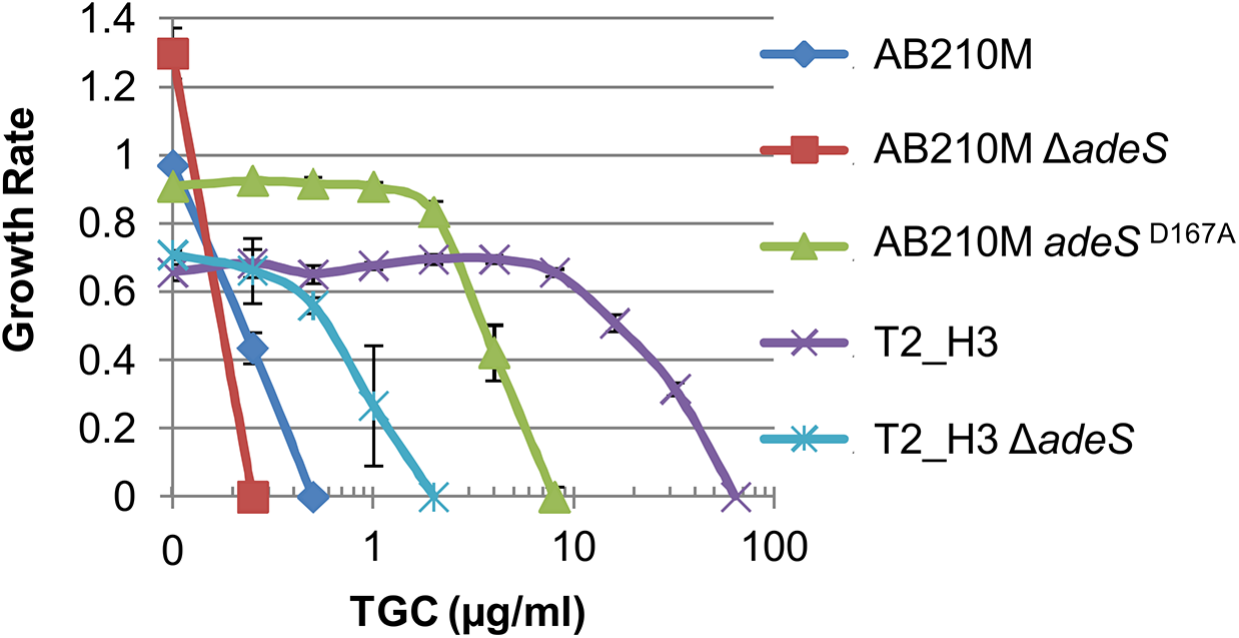
*adeS* mutations increase TGC MIC, but not to the level detected in bioreactor isolates. Strains were cultured in MHBII with increasing amounts of TGC to measure the growth rate. The MIC of each strain is the point where the growth rate equals 0. Error bars represent the 95% confidence interval.

To genetically determine if *adeS* point mutations convey the full extent of TGC resistance, we constructed strains containing a markerless deletion of *adeS* or the *adeS* ^D167A^ mutation in place of the native allele in AB210M. *adeS* was removed from AB210M and T2_H3, a bioreactor isolate with only 3 mutations: *adeS* ^D167A^, 19.6 kb deletion in contig 4 (including *gna*, WM39_01270), and a 4-bp intergenic deletion between uridylate kinase (WM39_12300) and *rrf* (WM39_12295). If nsSNPs in *adeS* alone provided high level resistance, we expected the TGC MIC of AB210M *adeS* ^D167A^ to be comparable to T2_H3, and the deletion of *adeS* from T2_H3 would result in an MIC equal to AB210M. The TGC MIC values demonstrated that the T2_H3 Δ*adeS* strain was more susceptible to TGC (2 μg/ml) than T2_H3 (64 μg/ml), but not as much as the ancestor AB210M (0.5 μg/ml) (Fig 6). Because AB210M *adeS* ^D167A^ (8 μg/ml) and T2_H3 Δ*adeS* had TGC MIC values between AB210M and T2_H3, at least one of the other mutations in T2_H3, the deletion including *gna* and/or the mutation upstream of *rrf*, must contribute to the high TGC MIC.

### Genomic analysis reveals both expected and novel families of genes associated with TGC resistance

Analyses of the polymorphic populations and isolates produced a list of 17 genes, including *adeS*, that may contribute to TGC resistance (Table 2 and S1 Text). Genes were selected for further analysis if they met at least one of the following criteria: 1) the Fisher Exact Test *p* value was < 0.001 for either the mixed population dataset or the clonal isolates; or 2) the gene contained the same nsSNP during both trials. If a gene can confer TGC resistance via a loss of function or slight change of function, we expect that many different mutations could have that effect, and we would detect the gene via the Fisher Exact Test. On the other hand, if there are relatively few positions in a gene that confer TGC resistance, we expect that the same mutation would be present in both trials. Therefore, we also used the criterion of a gene containing the same nsSNP in both trials.

**Fig 7:**
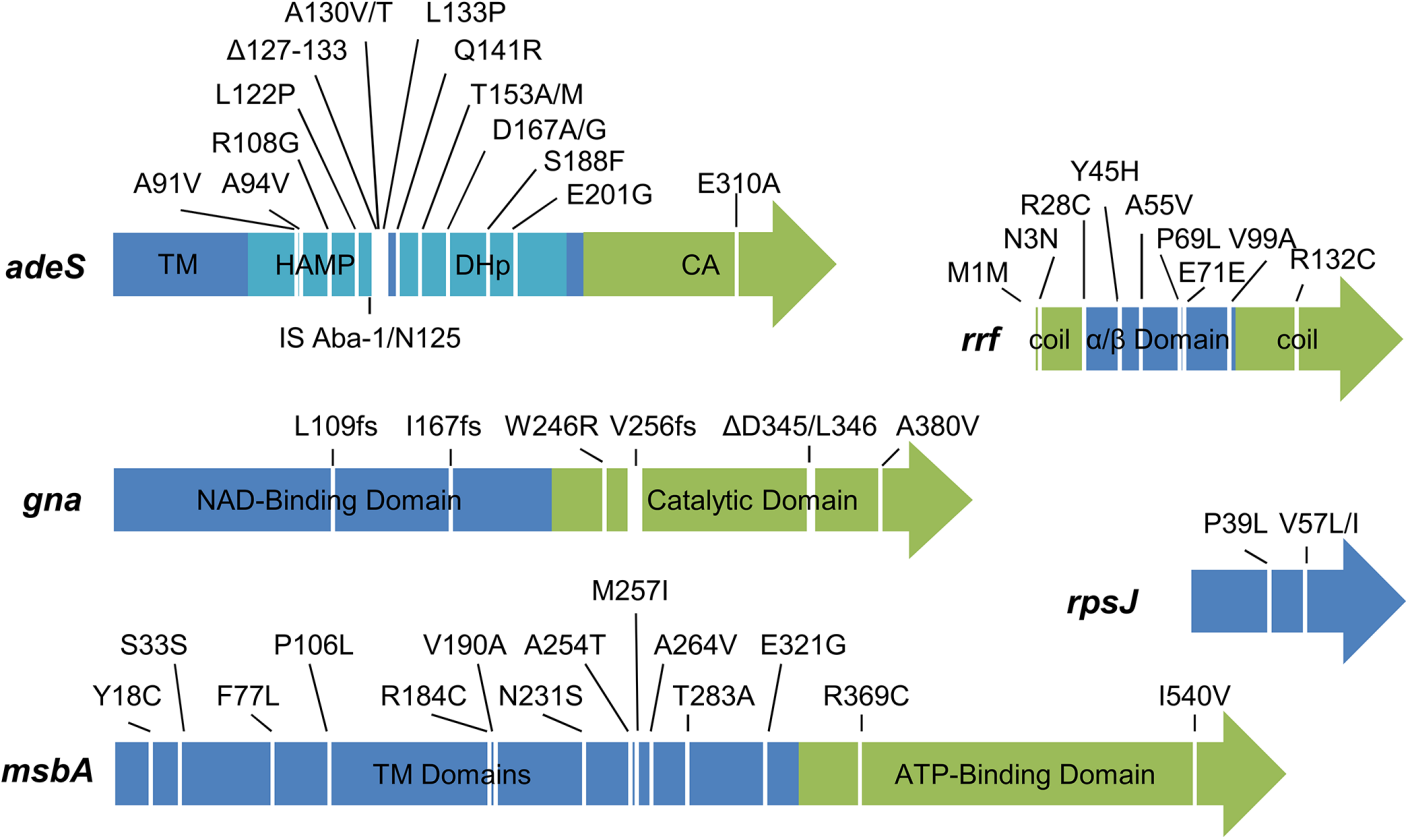
Mutations in genes that may reduce TGC susceptibility. Arrows represent the coding regions. The labels indicate the amino acid changes present in each protein. If a position was mutated to more than one residue, the two amino acids are listed, for example “A130V/T” indicates A130 was mutated to both a valine and threonine. Frameshift mutations are indicated by “fs”. For multi-domain proteins, the predicted domains are represented by different colors. HAMP, histidine kinase, adenyl cyclase, methyl-accepting protein, and phosphatase linker; DHp, dimerization and histidine-containing phosphotransfer domain; CA, catalytic and ATP-binding domain; TM, transmembrane motif.

From the list of 17 genes, we focused on five genes that may confer TGC resistance for further validation: *adeS*, *rpsJ*, *rrf*, *msbA*, and *gna* (Fig 7). Four genes (*adeS*, *rrf*, *msbA*, and *gna*) were mutated several times in many clones and therefore closest to the top right corner of the scatter plot (Fig 5D). In addition, *adeS*, *rrf*, and *msbA* have highly significant *p* values in both the population and clonal samples when the sequential Bonferroni correction was applied (Table 2). *rpsJ* was the fifth gene, and it was included because it contained identical nsSNPs in both trials and was previously shown to confer resistance to TGC [30,31]. Lastly, all of the sequenced isolates, including the two strains with only three and six mutations each, contained mutations in at least two of these genes. The other 12 genes are discussed in the S1 Text.

### Mutations in *adeS*, *rpsJ*, *rrf*, *msbA*, and *gna* appear at different times during the TGC adaptation

**Fig 8:**
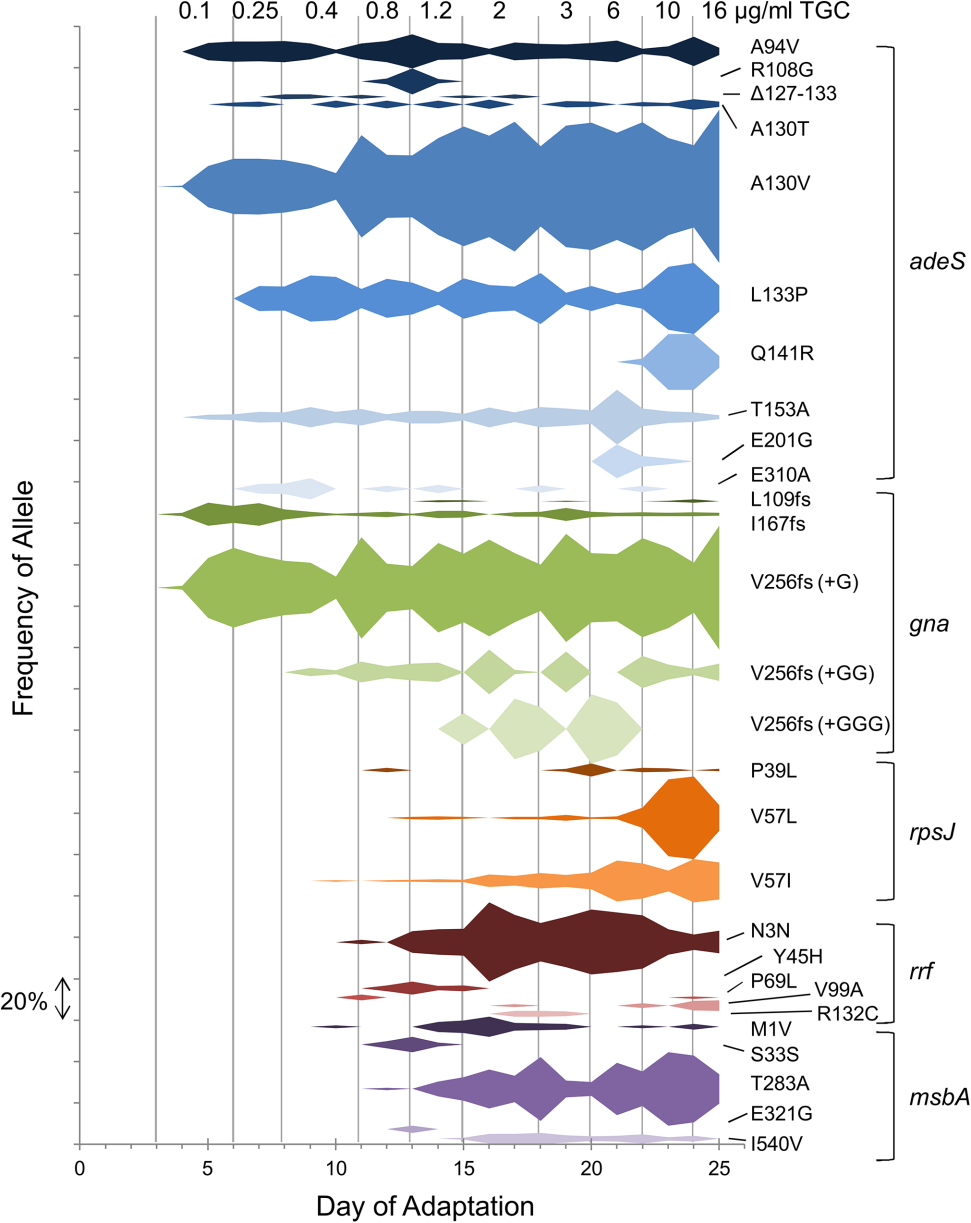
Frequencies of alleles associated with TGC resistance from Trial 1. Whole-genome sequencing data of mixed populations were collected every 24 hr. For clarity only mutations in the candidate genes *adeS*, *gna*, *rpsJ*, *rrf*, and *msbA* are shown. The mutations are grouped by gene. Each frequency is represented by the width (y-axis) of the colored object at the corresponding day (x-axis). The dashes on the left indicate a frequency of 20% and can be used as a scale to determine the frequency of a mutation on a specific day. Gray lines demark the different TGC concentrations in the culture.

To identify when mutations arose during selection and how the frequency of mutations changed over time, we performed whole-genome sequencing on mixed populations from each day during the first bioreactor adaptation trial. For simplicity, we plotted the data to track only the potentially adaptive mutations in *adeS*, *gna*, *rpsJ*, *rrf*, and *msbA* (Fig 8). Since we did not open the vessel to sample the biofilm communities directly, the population samples were largely comprised of planktonic cells, and thus not all mutations from the endpoint samples were observed during the time course. We reasoned that the earliest mutations would be the most important for adaptation. On the 4^th^ day of the trial when a sub-inhibitory concentration of TGC was present in the culture, mutations in *adeS* and *gna* were detected suggesting that the mutations provided a fitness benefit. New mutations in *adeS* continued to arise from days 4–8, but no other *gna* mutations were observed until later. Based on allelic frequency, the most successful mutations in *adeS* and *gna* were *adeS* ^A130V^ and an insertion of a G into codon 256 of *gna*, which caused a frameshift. By day 10 of the adaptation, mutations in *rpsJ*, *rrf*, and *msbA* were observed. Mutated alleles of *rpsJ*, *msbA*, and *rrf* did not reach the frequencies detected for *adeS* and *gna* suggesting that they played a necessary, but lesser, role in TGC adaptation.

### Identifying putative TGC-resistance genes in flask-adapted populations of *A*. *baumannii*

We reasoned that although our two bioreactor cultures had at least 8 independent, successful trajectories (S5 Fig), we could further confirm the relevance of our candidate TGC adaptive genes by increasing the number of independent, replicate populations [32]. Therefore, we adapted 10 populations of AB210M to TGC in a flask transfer experiment over 14 days by increasing the TGC concentration 1.5 times each day from 0.1 μg/ml to 19 μg/ml [33]. The resistance level of isolates at the end of the experiment ranged from 48 to >128 μg/ml TGC (data not shown).

We sequenced *adeRS*, *rpsJ*, *rrf*, *msbA*, and *gna* and amplified the *mutS* locus from at least two colonies on the last day of the experiment from each TGC evolved population and from a control population evolved in parallel, but without antibiotic (S2 Table). As observed in the bioreactor trials, IS15 D1 inserted into the *mutS* locus in 6 of the 10 populations. The control population did not contain mutations in any of the genes tested nor a deletion in the *mutS* locus. All populations exposed to TGC contained a mutation in *adeS* or in its cognate response regulator *adeR*. Two populations contained *rpsJ* mutations: population 7 had *rpsJ* ^V57I^ while population 10 contained *rpsJ* ^V57L^. Eight populations had mutations in the *rrf* gene or within 10 nucleotides upstream of the start codon. The gene encoding MsbA obtained seven mutations in six populations. Finally, *gna* frameshift mutations were identified in seven populations, and one population had a mutation 69 nucleotides upstream of *gna*. Taken together with our bioreactor results, the high number of populations containing mutations in *rpsJ*, *rrf*, *gna*, and *msbA* strongly suggests that these genes play a role in TGC resistance.

## Discussion

Hypermutating strains can arise during adaptation to an environment whether that environment is a human host [18,34,35] or a laboratory system [2,22,24]. In this study, we found that, as in the patient, a strain of *A*. *baumannii* evolved a hypermutator phenotype repeatedly while adapting to the front-line antibiotic tigecycline (TGC). We exploited the wealth of mutations generated by the hypermutator to explore the potential evolutionary trajectories that lead to TGC resistance. Six genes stood apart as the primary or secondary mechanisms to decrease susceptibility to TGC: *adeS*, *gna*, *rpsJ*, *rrf*, and *msbA*. One important aspect of our work is the discovery of new adaptive alleles that can confer high levels of TGC-resistance in conjunction with the previously characterized *adeS* family of mutations.

In complex ecologies where many competing microbial species are present and variable environmental conditions challenge the fitness of an organism, hypermutation will lead to rapid decline in fitness. However, in a sterile site, such as an indwelling medical device or within a wound, hypermutator strains can flourish as strongly adaptive mutations provide higher fitness to an antibiotic than the costs of rapidly accumulating deleterious mutations [4]. This is particularly true over the shorter time scale of a clinical infection or strong selection via antibiotic usage; therefore, it may not be surprising that hypermutator phenotypes have been observed for pathogens including *E*. *coli*, *P*. *aeruginosa*, and *S*. *enterica* causing chronic, persistent infections [1,34–38]. In addition, *A*. *baumannii* AB210, the ancestor for our studies, evolved into AB211, a TGC-resistant strain that contained an inactivated *mutS* via a large deletion, during an intra-abdominal infection in a patient receiving TGC. AB211 harbored several additional mutations including *adeS* ^A94V^ resulting in an overexpression of the AdeABC efflux pump likely conferring TGC resistance [18].

The unusually large number of independent events leading to the development of the hypermutator phenotype in the bioreactor populations suggests that it conferred a substantial benefit to the successful evolutionary trajectories. During the adaptation experiments, we identified 26 unique IS15 D1 insertions in the *mutS* region leading to its deletion (Fig 1). With the increased mutation rate, mutations in *adeS* and other adaptive alleles could arise more frequently and achieve success in the culture. It is important to point out that two of the clonal isolates (T2_F9 and T2_H3) did not evolve as hypermutators indicating that hypermutation is not required for successful adaptation to TGC and that the deleted region does not increase TGC resistance.

It is clear from our data and others that the most important adaptive changes to reduce TGC susceptibility occur through mutations in *adeS* that upregulate *adeABC*. All of our successful evolutionary trajectories included at least one mutation that likely increased *adeABC* expression. Mutations to *adeS* that lead to increased expression of *adeABC* have been observed with remarkable consistency in TGC-resistant clinical strains [18,26–28]. In addition, *adeS* knockout strains have shown a decrease in *adeABC* induction and loss of TGC resistance [27]. Recently, two groups determined the sequences of *adeS* from different clonal clusters of *A*. *baumannii* and detected several polymorphic sites and an insertion of IS Aba-1 [27,28]. Previous reports have identified mutations in *adeS* at the same positions as our cultures including A94, IS Aba-1 insertion at N125, A130, D167, T153, and R313 [14,17,26–28,39]. As shown in Fig 7, 13 of the 16 *adeS* mutations in our study affected amino acids 91–167 of the 357-residue protein. This region is the junction between the histidine kinase, adenyl cyclase, methyl-accepting protein, and phosphatase linker (HAMP) and dimerization and histidine-containing Phosphotransfer (DHp) domains [28]. Interestingly, the phosphorylated histidine is in the same region at residue 149. As a histidine kinase of a two-component system, AdeS likely autophosphorylates in the presence of an activating signal and then phosphorylates AdeR, the response regulator [12]. The mutations we identified suggest that the AdeS-AdeR phosphor-relay may be deregulated causing constitutive phosphorylation of AdeR. Though *adeS* is the most important TGC resistance gene, the TGC MIC of T2_H3 Δ*adeS* was elevated over the ancestor strain indicating that additional TGC resistance genes are present. This led us to examine further the mutations identified from the bioreactor samples for additional adaptive mutations.

In the present study, over 2500 mutations were present in individual isolates or mixed populations at greater than 5% frequency. These mutations affected 1465, or nearly 40%, of the coding regions in AB210M. Analyzing the mutations found in clones and mixed populations using Fisher’s Exact Test and presence of parallel evolved nsSNPs resulted in 17 candidate genes that can contribute to TGC resistance (Table 2). We further reduced the list of putative TGC resistance genes to genes with the most significant *p* values when applying the sequential Bonferroni correction for multiple tests, which resulted in five genes that had the highest potential to confer TGC resistance.

### Candidate TGC genes and proposed mechanisms of action in TGC resistance

MsbA is an essential ATP-binding cassette transporter (ABC-transporter) [40]. MsbA transports lipid A molecules from the inner leaflet of the inner membrane into the periplasmic side of the inner membrane. MsbA-like proteins in *Lactobacillus lactis* have been shown to confer resistance to antibiotics and toxic compounds [41]. Interestingly, overproduction of lipid A decreases the transport of antibiotics via MsbA [42,43]. Chen et al. recently reported a mutation in *msbA* in a TGC-resistant isolate of *A*. *baumannii* from a serial passage experiment [44]. When the authors overexpressed wild-type *msbA* in the mutant background containing *msbA* ^A84V^, the TGC MIC did not change leading them to conclude that *msbA* was not involved in resistance. Complementing a mutant allele with the native allele *in trans* is effective for loss-of-function mutations; however, the method does not necessarily provide insight into gain-of-function mutations. Since 12 of the 14 mutations in *msbA* from the bioreactor samples were located in the substrate-recognition and transmembrane portion of the protein, we speculate that the mutations broaden the specificity of the pump and facilitate efflux of TGC. Therefore, the *msbA* mutations likely were gain-of-function mutations, and *msbA* should be reexamined as a potential player in TGC resistance.

S10 is the ribosomal protein closest to the TGC binding pocket and is a general target for TGC resistance [33]. Previously, researchers have discovered S10 mutations which confer TGC and tetracycline resistance in *Neisseria gonorrhoeae*, *Enterococcus faecium*, *K*. *pneumoniae*, *Enterococcus faecalis*, *and E*. *coli* [23,30,31,33,45]. The mutations were located in the same extended loop (amino acids 53–60) as V57L and V57I found in Trials 1 and 2. We hypothesize that mutations in the S10 loop alter the TGC binding pocket of the ribosome resulting in a decrease in TGC affinity for the ribosome.

RRF, with the help of elongation factor Tu, recycles ribosomes by dissociating the large and small subunits after reaching a stop codon or if a ribosome stalls during translation [46]. Since TGC binds to the A site within the ribosome and prevents elongation of a polypeptide, RRF may play a role in dissociating TGC-bound ribosomes that appear to be stalled on the mRNA. RRF is an essential protein; therefore, the mutations are not likely to completely disrupt RRF function [47]. However, we predict that the mutations may decrease RRF function. One of the mutations we identified (RRF^R132C^) was shown in an earlier study to decrease RRF-dependent disassembly of ribosomes in *E*. *coli* [48]. In addition, two mutations were present at the beginning of the *rrf* gene that encodes RRF (M1V and N3N) and are likely to decrease translation from the *rrf* mRNA. The M1V mutation is a conversion of an ATG to GTG. GTG is an alternative start codon in *A*. *baumannii* and is predicted to be used by 172 of the 3741 protein coding genes. If the total amount or activity of RRF is reduced, then ribosomes stalled by TGC may be able to pause until the drug diffuses away from the binding site and proceed with translation rather than be recycled prematurely.

The *gna* gene resides within the K locus, a region encoding extracellular polysaccharide biosynthesis enzymes which play a role in constructing capsule or lipooligosaccharide (LOS) [49]. AB210M contains KL7, which is predicted to synthesize legionaminic acid (originally identified in *A*. *baumannii* strain TCDC-AB0715 [50]). Increased expression of the K locus was recently shown to decrease susceptibility to colistin and other peptide antibiotics [51], and work in *P*. *aeruginosa* demonstrated that certain polysaccharides can bind antibiotics [52]. The *gna* gene is one of 22 genes in this operon and converts UDP-*N*-acetyl-D-glucosamine to UDP-*N*-acetyl-D-glucosaminuronic acid or UDP-*N*-acetyl-D-galactosamine to UDP-*N*-acetyl-D-galactosaminuronic acid [49]. In the TGC-resistant bioreactor samples, *gna* contains several frameshift mutations likely disrupting its function. Interestingly, seven of the nine mutations in *gna* included repeat elements, such as homopolymer stretches and a 6 bp repeat. With the loss of Gna, the structure of the capsular polysaccharide or LOS would be modified. We speculate that changes in the extracellular polysaccharides could alter the rate of diffusion of TGC into the cell.

There does not appear to be a strong epistatic link between the TGC resistance genes. All the endpoint clones contained at least one *adeS* mutation, but there were many combinations of mutations in *rpsJ*, *rrf*, *msbA*, and *gna*. For example, strains were detected with a mutation in only one of the five candidate TGC resistance genes (i.e. T1_E12 *msbA*, T2_F9 *rpsJ*, T1_E8 *gna*). Likewise, there were clones with combinations of mutations in only two genes (i.e. T1_F6 *rpsJ* and *msbA*, T2_G7 *rrf* and *msbA*, T2_H3 *rrf* and *gna*, and T2_H4 *msbA* and *gna*). Additional work is needed to determine the contributions of each gene to TGC resistance and the epistatic relationships (if any) between the genes. It is possible that some of the changes correlated with adaptation to TGC may be compensatory for earlier mutations to improve fitness.

The evolutionary dynamics of emergent pathogens provide an interesting contrast to our more classical views of long-term evolution. It is clear from both theory and experimental evolution that hypermutators provide short-term gains but are essentially a dead end as deleterious mutations continue to accumulate. However, during strong selection and over shorter time scales, hypermutators can be highly successful and can lead to patient deaths. In this study we show that under selection to TGC, *A*. *baumannii* commonly evolves a hypermutator phenotype that illustrated both a common clinical mechanism for genetic adaptation and permitted a comprehensive survey of the successful evolutionary trajectories. We identified known alleles leading to TGC resistance as well as new ones not yet discovered clinically but that may emerge in the near future.

## Materials and Methods

### Bacterial strains and culturing conditions

*A*. *baumannii* strains AB210M and AB211 were obtained from Dr. Michael Hornsey at Queen Mary’s University London (S3 Table). *A*. *baumannii* were cultured in Luria Broth (LB) or Mueller Hinton Broth supplemented with 20 mg/ml CaCl_2_ and 10 mg/ml MgCl_2_ (MHBII) at 37°C. *E*. *coli* strain DH5α was used for cloning purposes and was cultured in LB at 37°C. When needed, 50 μg/ml kanamycin (KAN) was added to the medium. Tigecycline (TGC) was prepared fresh or frozen at -20°C and added to medium freshly prepared or medium frozen at -80°C to prevent aeration.

### Adaptation of AB210M to TGC in a bioreactor using metabolic control

We chose to use a continuous culture to evolve TGC resistance in AB210M. For our purposes, continuous cultures in a bioreactor offer several benefits over traditional flask transfer experimental evolution including more generations per day, larger and more genetically diverse populations, no selection for shorter lag times or increased survival in stationary phase, a constant level of non-limiting nutrients, constant antibiotic concentration and conditions that favor the evolution and persistence of strong biofilm forming communities. We completed two adaptation experiments (Trial 1 and Trial 2), which gave us a measure of replication suitable for the population size and diversity detected within each culture.

We operated the continuous culture as a turbidostat (culture with a constant turbidity) with one important exception: biofilm developed on the surfaces within the bioreactor which increased the total population size. Formation of the desired and aggressive biofilm precludes using an internal probe to measure optical light scattering as a means of estimating population density, and therefore, we regulated the bioreactor population by measuring the production of CO_2_ as a proxy for overall metabolism. The inflow of medium was dynamically controlled to maintain OD_600_ ~ 0.44 (~10^8^ cfu/ml) in 300 ml LB. The culture turbidity was measured offline manually (MacFarland Densitometer, Grant Instruments, Cambridgeshire, UK) and then correlated to the exhausted CO_2_ concentration (Magellen Biotech, Hertfordshire, UK). Inflow pumps activated when the CO_2_ concentration increased above a set point.

For the first three days of each trial, the populations were cultured without TGC to provide an opportunity for the cells to adapt to the bioreactor and establish biofilms. To begin the selection, 0.1 μg/ml TGC (less than half the MIC of AB210M) was added to the culture medium. Daily, we measured the MIC of the culture and increased the TGC concentration only when the growth rate of the population was not affected by the new drug concentration. For Trial 1, the following drug titration was employed: 0.1, 0.25, 0.4, 0.8, 1.2, 2, 3, 6, 10, and 16 μg/ml TGC. Trial 2 followed a similar titration; however, instead of 6 and 10 μg/ml, we added 5 and 8 μg/ml TGC. Using sub-MIC antibiotic concentrations leads to incremental changes in the MIC of the population [21,23].

Daily samples contained primarily planktonic culture and were collected every 24 h. The 30 ml samples (approximately 10% of the culture) were stored in 30% glycerol at -80°C. We harvested biofilm samples from the borosilicate glass walls and the stainless steel frame of the bioreactor at the end of each trial.

### Phenotypic assays of bioreactor-derived strains

To identify sub-populations within the bioreactor, we performed several phenotypic assays on endpoint isolates including agar-dilution TGC MIC assays with or without the efflux pump inhibitor 1-(1-naphthylmethyl)-piperazine (NMP, Sigma-Aldrich), growth curves with and without 16 μg/ml TGC, swarming assays, KAN susceptibility, and colony morphology. Using the protocol of Wiegand et al. [53], we set up agar-dilution TGC MIC assays with LB agar containing two-fold dilutions of TGC ranging from 0.25 to 128 μg/ml in the presence or absence of 64 μg/ml NMP. Isolates were cultured in LB for 24 hr and spotted onto plates. A similar method was used to determine susceptibility to KAN using LB plates containing 50 μg/ml KAN. For the swarming assays, 1 μl stationary phase cultures were spotted onto LB containing 0.3% Difco Agar in 24-well plates. The diameters of the colonies after incubating for 18hr were compared to the ancestor. To measure colony morphology, we scored each colony as it appeared on an LB plate (see S1 Text).

With the phenotypic data, we performed hierarchical and k-means clustering using R via RStudio [54,55] to detect distinct groups of strains (see S1 Text). These data suggested that there were as few as 6 and as many as 10 sub-populations (S3 Fig and S4 Fig). We selected single colonies to represent these sub-populations for whole genome sequencing.

### Whole genome sequencing and analysis

The ancestral strain (AB210M), mixed populations, and clonal samples from the bioreactor trials were sequenced. Genomic DNA was extracted directly from the frozen samples using the MO BIO Ultraclean Microbial DNA Extraction Kit (MO BIO, Carlsbad, California). For the biofilm samples, DNA was extracted using the PowerBiofilm DNA Isolation Kit (MO BIO). We prepared barcoded libraries with the Nextera XT kit (Illumina, San Diego, California) and sequenced on the Illumina HiSeq Platform resulting in 101 or 93-bp paired-end reads with at least 100x or 300x coverage for the clonal and population samples, respectively. All data were deposited to the Sequence Read Archive under BioProject #PRJNA279596.

The AB210M reference sequence was constructed by modifying the AB210 genome (NCBI accession #AEOX00000000, see S1 Text). The AB210M genome consists of 72 contiguous sequences (contigs) containing 4.06 Mb. The AB210M reference genome was deposited into NCBI as LAPU00000000 and genes are numbered WM39_00005–WM39_19210.

All clonal samples and mixed populations were aligned to the AB210M reference genome using breseq v.024rc7 [56]. The clonal samples were analyzed with the default settings, whereas mixed populations were run with the polymorphism (-p) and new junction options (-j2). Outputs were combined using the COMPARE function within breseq’s gdtools to make two different tables: metagenomic endpoint populations and TGC resistant endpoint isolates. For simplicity, mutations from all samples (Table 1: daily metagenomic samples, endpoint populations, and endpoint isolates) were compiled and listed in S1 Table. For polymorphic samples, breseq calls mutations that are greater than 0.3%; however, we only retained a mutation if it was present in a clonal sample or the maximum frequency for the mutation was greater than or equal to 5%.

### Statistical Analysis of mutated genes

The Fisher’s Exact Test was used to determine if a gene was mutated more frequently than expected if mutations were distributed randomly. The input for the Fisher’s Exact Tests were the number of mutations in the dataset (896 for polymorphic populations and 1712 for isolated clones), genome size (3.98 Gb), number of mutations in that gene and length of the gene. To determine if *p* values were significant, we used a sequential Bonferroni correction for multiple tests. After ranking the *p* values from largest to smallest, we compared the *p* value to 0.05 divided by the rank of the *p* value. If the *p* value was less than the correction, the result was deemed significant. For the polymorphic populations, which contained 626 mutated genes, 8.04 x 10^−5^ was the cutoff for significance. The threshold for the isolated clones which contained 1093 mutated genes was < 4.64 x 10^−5^.

### Construction of Δ*adeS* and *adeS* ^D167A^ strains

We constructed plasmids to generate an *adeS* point mutation and *adeS* null mutation in AB210M and bioreactor isolate T2_H3. We obtained pMo130-TelR from Chua Kim Lee at Yong Loo Lin School of Medicine, National University of Singapore via Addgene [57]. To amplify *adeS* ^D167A^, we used genomic DNA from T2_H3 and primers RSH24 and TH496 which contain BamHI and NotI restriction sites, respectively (S4 Table). The *adeS* deletion vector was created with RSH24 and TH503 to amplify *adeR* and TH502 and TH496 to amplify *adeT*. The resulting products were fused via PCR-SOEing [58]. The PCR products were digested with BamHI and NotI and ligated into pMo130-TelR digested with the same enzymes to make pMo130-TelR *adeS* ^D167A^ or pMo130-TelR Δ*adeS* (S3 Table). After transforming *E*. *coli* DH5α and sequencing the plasmids, the vectors were electroporated into competent AB210M or T2_H3 prepared according to the protocol of Aranda J et al. [59]. Transformants had integrated the plasmid into the *adeRST* locus and were selected on LB agar containing 30 μg/ml potassium tellurite. Transformants were pooled and passaged 1–4 times in LB with 10% sucrose to facilitate homologous recombination and excision of the plasmid. The entire *adeRST* locus was sequenced to ensure that only the desired mutation was present. In addition, we confirmed by PCR that the *mutS* region was intact and the additional mutations in T2_H3 were still present.

### MIC growth rate assay

The TGC MIC values of strains encoding mutant *adeS* alleles were determined using growth rate assays as explained previously [60]. Briefly, 3–5 colonies of each strain were cultured in 3 ml MHBII until turbid. The cultures were diluted to approximately 1 x 10^6^ CFU/ml and combined in a 96-well plate with MHBII containing two-fold dilutions between 0.125 and 128 μg/mL TGC. The plate was incubated at 37°C, and the OD_600_ was measured every 5 min for 14 hours. Growth rate and lag time were calculated for each well, and the MIC for each strain was equal to the TGC concentration when the growth rate was zero.

### Flask transfer adaptation of AB210M to TGC

To increase the number of replicate cultures and provide additional experimental validation for our findings from the bioreactor, we adapted 10 cultures of AB210M to TGC using the classic flask transfer technique [61]. On the first day, we inoculated all 10 cultures with the same stationary-phase culture of AB210M. After incubating for 24 h, 100 μl of culture was transferred to a new test tube containing 10 ml LB with 0.1 μg/ml TGC. The next day, 100 μl of culture was transferred to 10 ml LB with 1.5 x the TGC concentration from the previous day. The passages continued until day 14 when the cultures contained 19 μg/ml TGC. In parallel with the TGC cultures, we cultured AB210M without antibiotic.

Putative TGC-resistance genes were sequenced from individual colonies at the end of the flask transfer experiments. Genes were amplified from individual colonies from each TGC-adapted population and the control population and subjected to Sanger sequencing (See S4 Table for the list of primers). Reads were compared to the AB210M reference sequence.

## Supporting Information

S1 Fig Sequencing data revealed differences in spatial populations. Plots show the frequencies of mutations in mixed population samples from different sampling sites. If bacteria in each population moved between niches or there was parallel evolution between niches, the points would fall near a line y = x.
(TIF)

S2 Fig Large deletions sweep through the population rapidly. The frequencies of large deletions were determined by the read coverage of the contig compared to the average coverage depth across the entire genome. Note, the deletion of AEOXM025 was associated with the insertion of IS15 D1 into the *mutS* locus, and the frequency of deletion of AEOXM025 is the sum of all the insertions in *mutS*.
(TIF)

S3 Fig Phenotypic clustering of endpoint isolates indicates the underlying genetic diversity within the populations. 90 isolated colonies from the end of each bioreactor trial were subjected to phenotypic assays resulting in 11 different quantitative and categorical values. Heat maps show the scaled values for each colony from (A) Trial 1 or (B) Trial 2. The values are represented by the intensity of the green color. The colonies and assays are grouped based on the values.
(TIF)

S4 Fig Sequenced Trial 1 and Trial 2 clonal isolates had very different phenotypes. The table lists the sampling site, colony morphology, TGC MIC in μg/ml, presence of NMP inhibition, swarming motility, and kanamycin resistance. Plus (+) indicates that the phenotype was observed. The bar graph displays the replication rates in LB with 16 μg/ml TGC (red) or in LB alone (blue). The replication rates were normalized to the ancestor AB210M in LB. Error bars represent one standard deviation.
(TIF)

S5 Fig Phylogenetic trees of isolates from Trials 1 and 2 show the diversity within each population. Trees were created with the nsSNPs using PHYLIP v3.695 with the bootstrapping and maximum likelihood programs.
(TIF)

S1 Table Mutations from all samples from the bioreactor trials. Whole-genome sequencing data were compared to the AB210M ancestral genome and aligned using breseq. There were 2552 mutations in at least one sample that passed our quality control filters (100% frequency in a clonal isolate or the maximum detected frequency was greater than 5%). The table lists every mutation and pertinent information including genome position, contig, contig position, gene, and the frequency of the mutation in a specific sample.
(XLSX)

S2 Table Putative TGC resistance genes mutated in bioreactor and flask adaptation experiments. Candidate TGC resistance genes were Sanger sequenced from 10 populations adapted to TGC via flask transfer along with one control, no drug population. The mutations present in at least two colonies are listed. Several populations were polymorphic at a locus which is indicated by multiple mutations present or a mutation and “wt” indicating that a portion of the population contained the wildtype sequence. Data for Bioreactor Trials 1 and 2 indicate the number of mutations detected in endpoint samples from that trial.
(XLSX)

S3 Table Strains and plasmids used in this study. (XLSX)

S4 Table Primers used in this study. (XLSX)

S5 Table Genomic Differences between AB210 and AB210M. **AB210M contains 50 mutations not present in AB210.** The mutations are listed along with the genome positions and gene number (locus ID from patricbrc.org).
(XLSX)

S6 Table Mutations in *ade* family genes. Here, we list the *ade* family genes that contained at least one mutation in a sample.
(XLSX)

S1 Text Supplemental Text. The text contains additional details about the differences between AB210 and AB210M, the phenotypic assays and clustering analysis, identification of the large deletions, additional genes that passed the bioinformatic filters, mutations in the *ade* gene family, and mutations in other TGC resistance genes.
(DOCX)
