# Supplementary figures and images for: Acinetobacter baumannii Repeatedly Evolves a Hypermutator Phenotype in Response to Tigecycline That Effectively Surveys Evolutionary Trajectories to Resistance
Source: PLoS One. 2015 Oct 21;10(10):e0140489. doi: 10.1371/journal.pone.0140489 (PMC4619398; doi:10.1371/journal.pone.0140489)

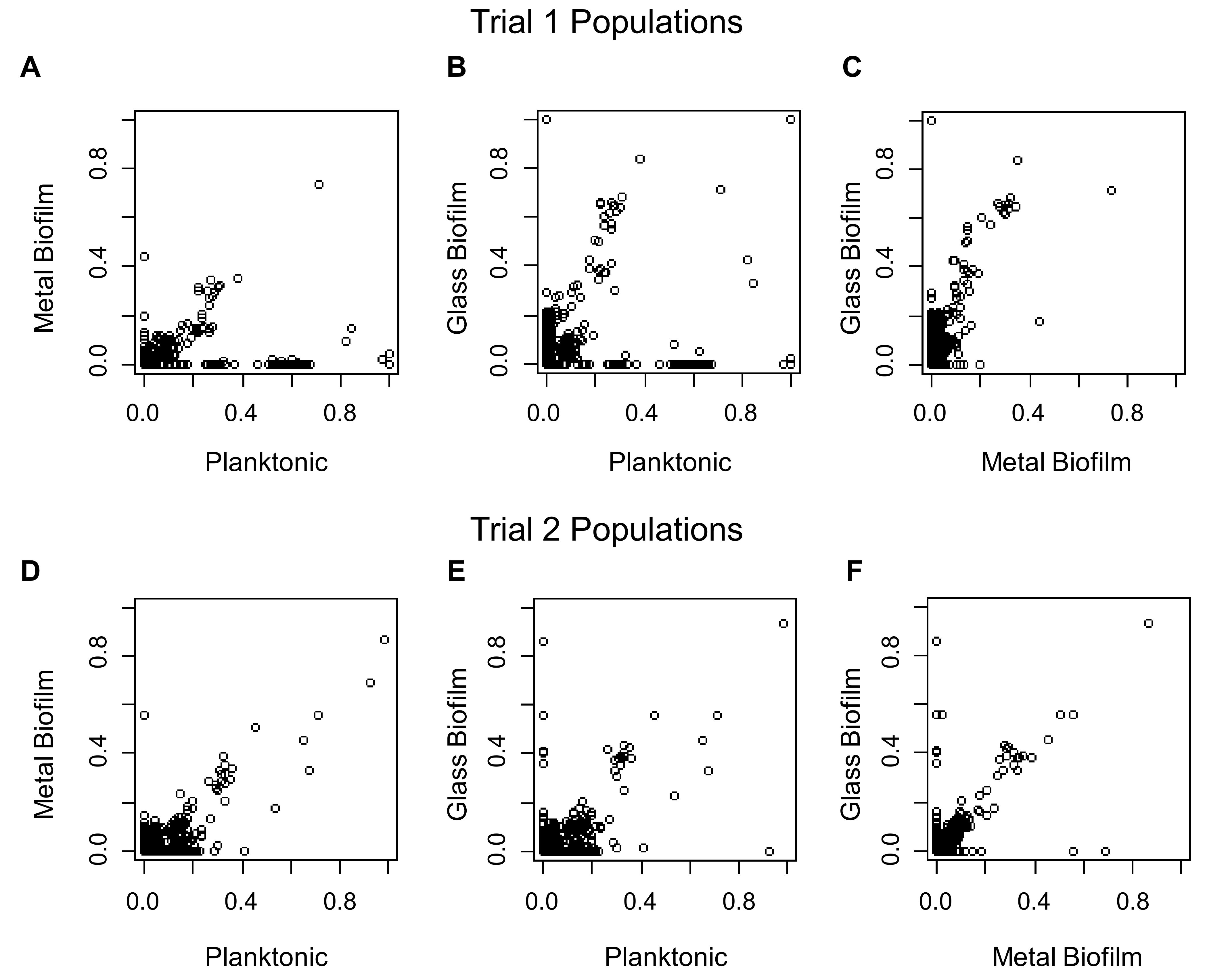

Supplement: S1 Fig — Plots show the frequencies of mutations in mixed population samples from different sampling sites. If bacteria in each population moved between niches or there was parallel evolution between niches, the points would fall near a line y = x. (TIF) [file pone.0140489.s001.tif]

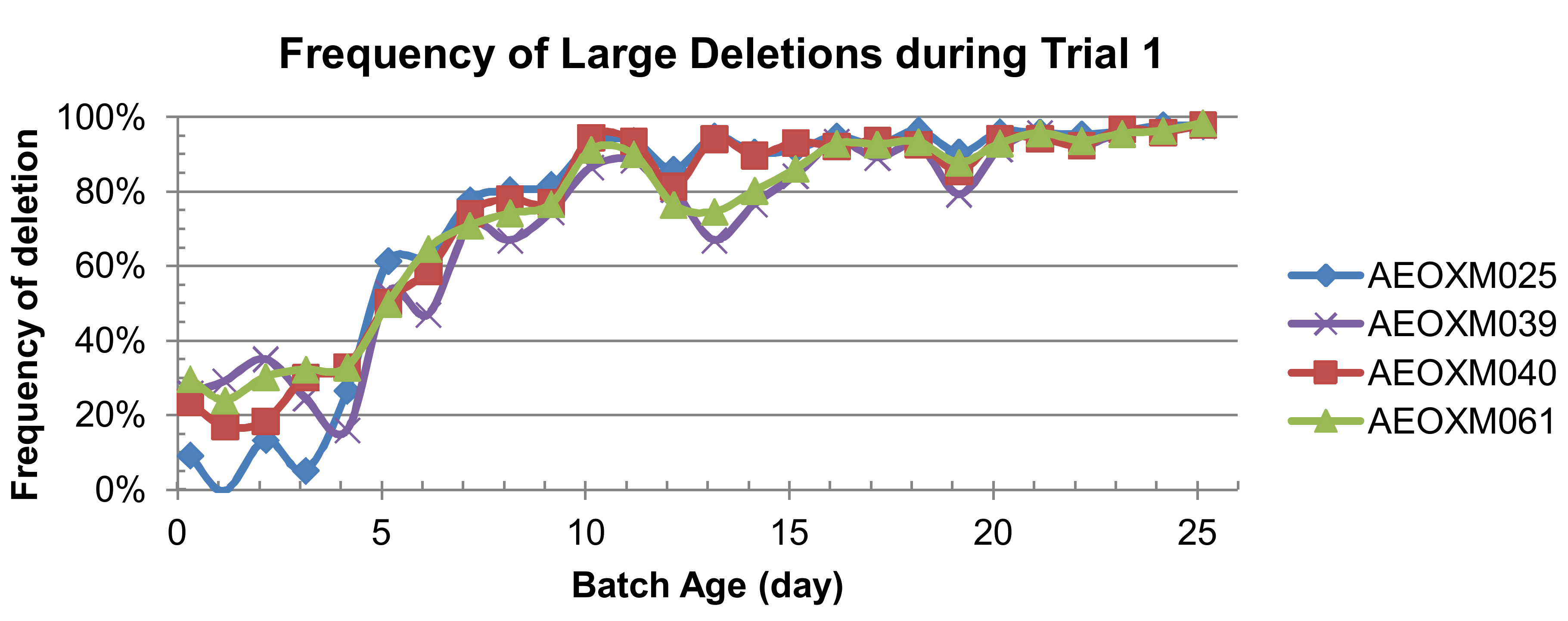

Supplement: S2 Fig — The frequencies of large deletions were determined by the read coverage of the contig compared to the average coverage depth across the entire genome. Note, the deletion of AEOXM025 was associated with the insertion of IS15 D1 into the mutS locus, and the frequency of deletion of AEOXM025 is the sum of all the insertions in mutS. (TIF) [file pone.0140489.s002.tif]

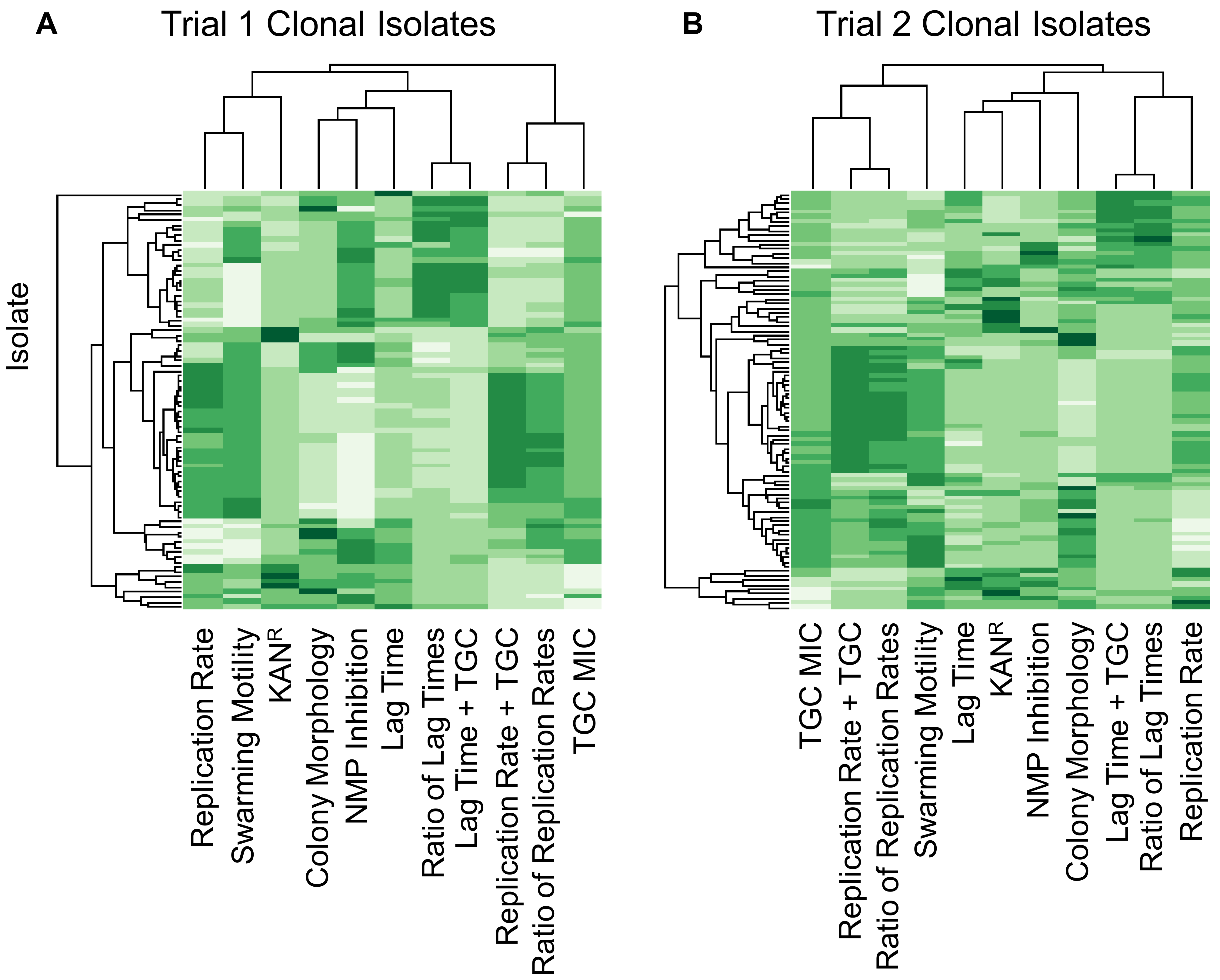

Supplement: S3 Fig — 90 isolated colonies from the end of each bioreactor trial were subjected to phenotypic assays resulting in 11 different quantitative and categorical values. Heat maps show the scaled values for each colony from (A) Trial 1 or (B) Trial 2. The values are represented by the intensity of the green color. The colonies and assays are grouped based on the values. (TIF) [file pone.0140489.s003.tif]

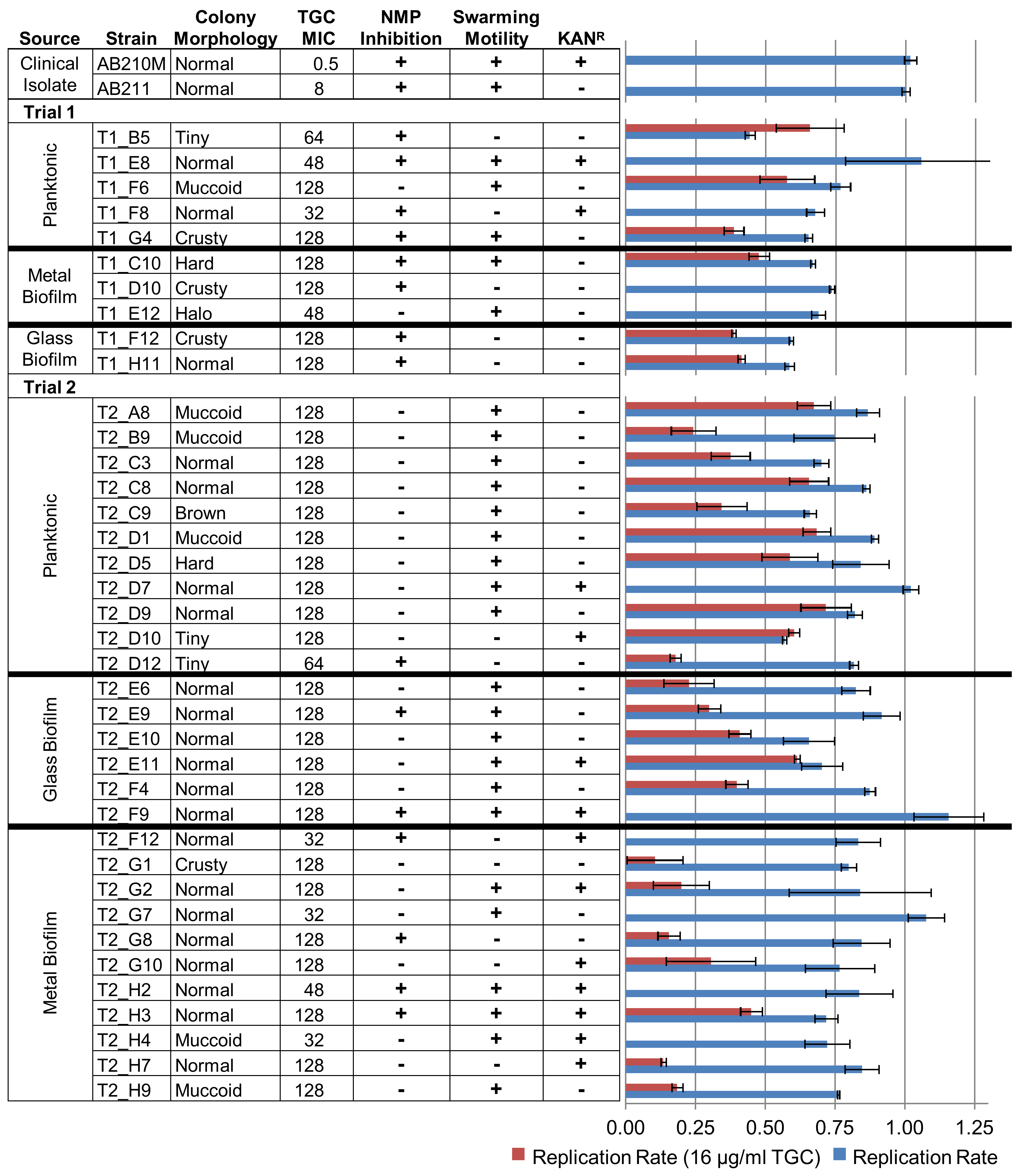

Supplement: S4 Fig — The table lists the sampling site, colony morphology, TGC MIC in μg/ml, presence of NMP inhibition, swarming motility, and kanamycin resistance. Plus (+) indicates that the phenotype was observed. The bar graph displays the replication rates in LB with 16 μg/ml TGC (red) or in LB alone (blue). The replication rates were normalized to the ancestor AB210M in LB. Error bars represent one standard deviation. (TIF) [file pone.0140489.s004.tif]

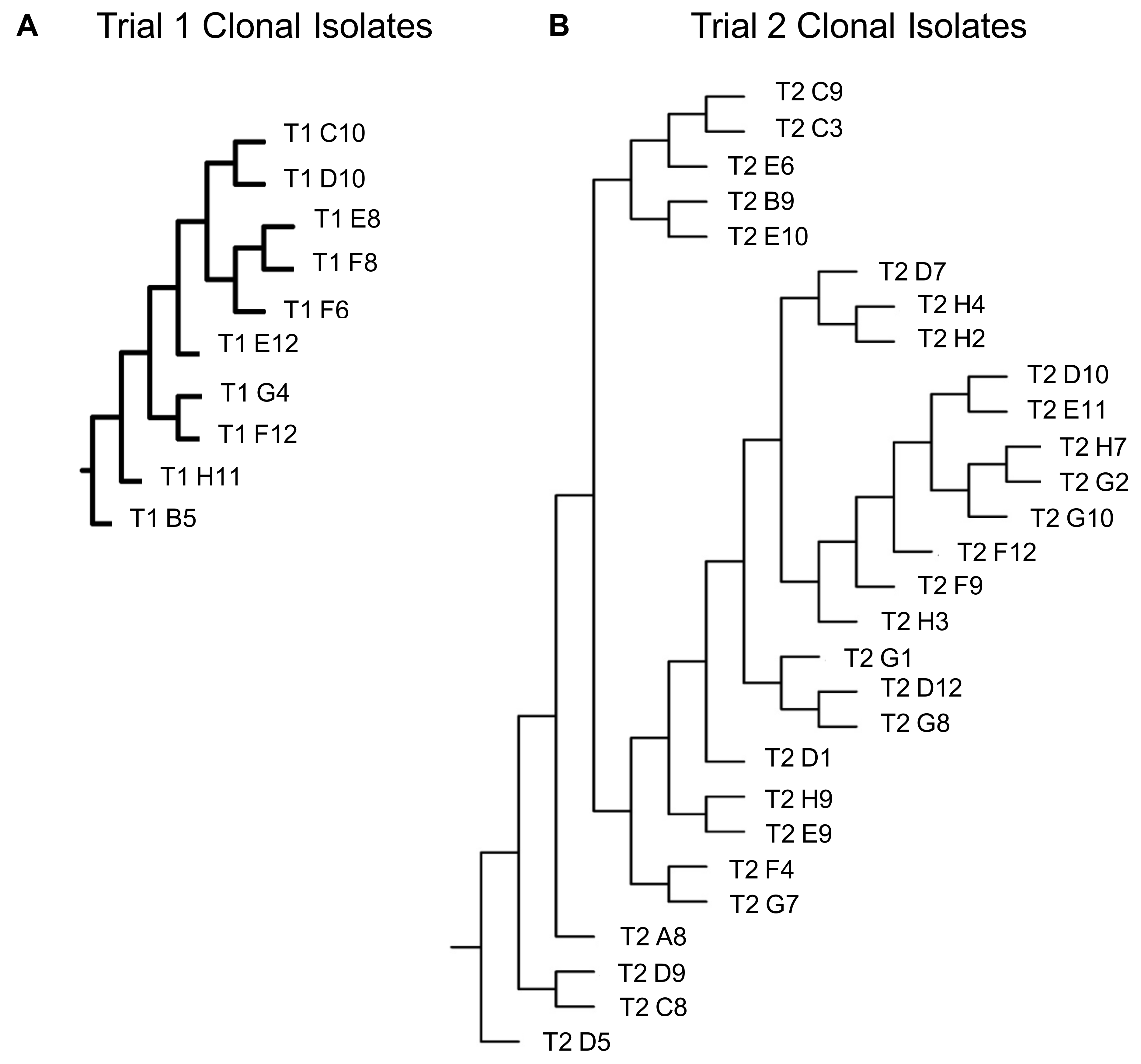

Supplement: S5 Fig — Trees were created with the nsSNPs using PHYLIP v3.695 with the bootstrapping and maximum likelihood programs. (TIF) [file pone.0140489.s005.tif]
