# Supplementary material for: Acinetobacter baumannii Repeatedly Evolves a Hypermutator Phenotype in Response to Tigecycline That Effectively Surveys Evolutionary Trajectories to Resistance
Source: PLoS One. 2015 Oct 21;10(10):e0140489. doi: 10.1371/journal.pone.0140489 (PMC4619398; doi:10.1371/journal.pone.0140489)
Supplement: S1 Text — The text contains additional details about the differences between AB210 and AB210M, the phenotypic assays and clustering analysis, identification of the large deletions, additional genes that passed the bioinformatic filters, mutations in the ade gene family, and mutations in other TGC resistance genes. (DOCX) [file pone.0140489.s012.docx]

**S1 Text**

*Genomic differences between AB210 and AB210M*

The ancestor for our study was AB210M, a strain derived from AB210, a TGC-sensitive clinical isolate [17]. During the studies presented herein, resequencing the ancestor revealed 49 mutations compared to the AB210 reference sequence (NCBI accession #AEOX00000000 [18]). The mutations included an nsSNP in *mutL*, 10 indels, 6 deletions that spanned the entire length of certain contigs, and 32 intergenic mutations (S5 Table). Three of the deleted contigs were pACICU2, a 65-kb plasmid present in AB210. Due to the mutations in our sample of AB210, we renamed the strain AB210M to avoid confusion with the original work.

To construct the AB210M genome, we modified the AB210 reference sequence. First, the AB210M reads were aligned to the AB210 reference genome via breseq v0.24rc7 [56]. Using the gdtool APPLY within breseq, we altered the AB210 genome with the mutations present in AB210M. Next, we used the new junction evidence from breseq to join several contigs. Finally, we used Mauve (v2.3.1, [63]) and the *A. baumannii* TYTH-1 genome to reorder the contigs placing AEOXM001-051 (3.9 of 4.06 Mb) in the proper order.

*Phenotypic clustering indicates genetic differences of T1 and T2 isolates*

Based on our previous experiments, single colonies must be assayed to understand the genetic heterogeneity found at the end of bioreactor adaptation trials [21,23]. Therefore, we isolated 90 colonies from each bioreactor trial, performed phenotypic assays, and then divided colonies into various clusters. At the end of the adaption trials, samples from each sampling site were diluted and spread onto LB plates. After 18-24 hr incubation at 37°C, colonies were selected in a biased fashion to represent all colony morphologies present (see below). The selected colonies were suspended in LB medium, cultured for 4-6 hr, mixed with glycerol to a 30% w/v final concentration and then aliquotted into 96-well plates for storage at -80°C. Each colony was measured using 11 assays that were performed at least three times with AB210M and AB211 serving as controls.

Colony Morphology: Colonies had a variety of colony morphologies, including normal, muccoid, crusty, hard, tiny, halo, and brown. “Muccoid” colonies had a large amount of gel-like material that would extend from the colony as it was picked with an inoculating loop. “Crusty” colonies broke into clumps when touched with a loop. “Hard” colonies were harder to disturb and generally came off the plate as a complete colony. The “tiny” colonies were much smaller than the ancestor (“normal”). Finally, the appearance of the colony on the plate was “brown” or left a white “halo” when the colony was lifted from the plate. The colony morphologies were stable for at least two passages on non-selective plates.

TGC Minimal Inhibitory Concentration (MIC): We assessed the TGC MIC for each colony. After culturing the clonal strains in 1 ml LB for 24 hr, they were spotted onto LB agar containing two-fold dilutions between 1 and 128 µg/ml TGC. Growth of each colony was assessed after 20 hr. Colonies had an MIC between 32 µg/ml and >128 µg/ml TGC.

Effect of NMP: In conjunction with the TGC MIC assay, we set up LB agar plates that contained increasing concentrations of TGC plus 64 µg/ml NMP, which is an efflux pump inhibitor. NMP was resuspended in 100% ethanol at 64 mg/ml. If the TGC MIC was different when NMP was added, the colony was affected by NMP indicating that efflux pumps played a major role in resistance for that colony.

Kanamycin Resistance: The initial clinical isolate, AB210, was resistant to kanamycin (KAN); however, the TGC-resistant isolate AB211 lost the KAN resistance in the patient [17,18]. Due to this change in AB211, we tested the TGC-adapted strains with 50 µg/ml KAN. We spotted the strains onto an LB plate with 50 µg/ml KAN and incubated the plate for 20 hr. Only 10 T1 isolates and 20 T2 isolates retained their resistance to KAN. As detailed by Horney and colleagues and below, the KAN resistance gene is on a transposon that was deleted in several strains [18].

Swarming Motility: *A. baumannii* exhibits swarming motility on medium with a low agar concentration [64]. We assessed swarming motility by spotting 1 µl stationary phase culture onto LB with 0.3% Difco agar. After incubation at 37°C for 18-20 hr, we compared the colony size of each strain to the AB210M ancestor. If the colony size was equal to or greater than AB210M, the strain was scored as motile.

Replication Rate and Lag Time in the presence or absence of 16 µg/ml TGC: We measured replication rate and lag time using a 96-well based assay in a microplate reader [60]. Stationary phase cultures were diluted 100-fold in LB with or without 16 µg/ml TGC, which was the highest concentration of TGC in the bioreactor. The cells were cultured for 20-24 hr with optical density measurements taken every 5 min. We calculated lag time and maximum replication rate for each strain. In addition, we determined the changes in replication rate and lag time when adding TGC to the medium by dividing each value in the medium with TGC by the value without TGC (i.e. Lag Time with TGC / Lag Time without TGC). These assays provided six measurements used to differentiate the strains.

MIC values, replication rates, and lag times were quantitative measures, whereas inhibition by NMP, swarming ability, colony morphology, and KAN resistance were scored categorically. The categorical values were scored as 0 (no change to TGC MIC when NMP was added, no swarming motility, or susceptible to KAN) or 1 (NMP changes MIC, swarming motility present, or resistant to KAN). The averaged values or modes from the independent trials of each assay were tabulated for the quantitative and categorical assays, respectively. Using R, we scaled the quantitative data by calculating the mean for each assay and then representing each value as the number of standard deviations from the mean. A heatmap of the results for each colony shows the diversity within each bioreactor trial (S3 Fig.). Using the phenotypic data, we performed hierarchical clustering and k means clustering to find subpopulations in the isolates from each adaptation trial. After sequencing the 10 isolates from Trial 1 and discovering the hypermutator phenotype, we decided to sequence 28 isolates from Trial 2. The additional isolates allowed us to discover rare alleles. The phenotypic data for the sequenced isolates is reported as figure S4.

After sequencing the isolates and creating a phylogenetic tree, the 10 clones from Trial 1 group into four sub-populations. The Trial 2 clones were more diverse and were present in six major subgroups encompassing between three and seven strains each (S5 Fig.).

*Large Deletions in T1 and T2 strains*

Many bioreactor isolates and mixed populations contained four large deletions making the strains deficient in several biochemical pathways and susceptible to KAN. The four whole-contig deletions were AEOXM025 (35 genes, 42.6 kb), AEOXM039 (17 genes, 17.9 kb), AEOXM040 (5 genes, 3.6 kb), and AEOXM061 (1 gene, 1.5 kb). For simplicity, contigs are listed by the last two numbers (i.e. AEOXM025 = contig 25). 36 of the 38 clones have the deletion in contig 25 which was linked to the partial deletion of contig 24 that included *mutS*. The deletions of contigs 39, 40, and 61 appeared to be linked as 25 isolates had mutations in all three regions. However, 3 isolates lacked contig 40 but contained contigs 39 and 61 suggesting that contigs 39 and 61 were adjacent and more closely linked.

Contigs 39 and 40 were adjacent to one another when the contigs were aligned to the closed *A. baumannii* TYTH-1 genome. IS15 D1 sequences flank the junction between contigs 38-39, 39-40, and 40-41. However, the insertion element was in the reverse orientation in the junction between contigs 38-39 and 39-40 and the forward orientation between contigs 40-41. Contig 39 matched region of deletion 3 (ROD3) in AB211 (clinical TGC-resistant isolate) reported by Hornsey and colleagues [18]. Contigs 40 and 61 were also deleted in AB211; however, they were not reported.

Contig 40 encoded five genes, none of which were predicted to participate in antibiotic resistance. Contig 61 contained an aminoglycoside 3’-phosphotransferase (*aph3ia*) and IS15 D1 repeat sequences. The genes encoded within contigs 25 and 39 were reported by Hornsey et al. [18] and summarized here. Contig 25 encoded only a few antibiotic resistance genes, there were transcription regulators, ion channels/transporters, and a Type VI secretion system. In contrast, contig 39 was a class 1 integron with many antibiotic resistance genes. The sequence was predicted to encode resistance to macrolides, aminoglycosides, and sulfonamides.

We were surprised to observe the deletions very early during adaptation (S2 Fig.). Using the read coverage of the contigs for the samples collected each day from Trial 1, we estimated the frequency of each deletion in the bioreactor population. The loss of the contigs was detected as early as the first sample (Day 1) collected 7 hours after inoculating the culture, but rose to near fixation (98%) by Day 10. Contigs 39, 40, and 61 have nearly identical frequencies with the exception of contig 40 during days 13 and 14. The separation of contig 40 from contigs 39 and 61 support the hypothesis that contigs 39 and 61 were more closely linked than contig 40.

Two clones from Trial 2 had additional large deletions. T2_G8 contained deletions in contigs 25, 39, 40, and 61 similar to other strains, but it also lacked an internal segment of contig 29 (position 119,008 – 154,706; 53 genes; 35.7 kb). Out of 53 genes in this region, there were 38 hypothetical genes. Many of the remaining genes were putative phage-related genes: phage tail subunits, Cro/CI transcriptional regulator, and phage terminase subunits. Therefore, this region appeared to be a dormant phage. T2_H3 contained a 19.6 kb, 19-gene deletion within contig 4. As described in the main text, this region encoded extracellular polysaccharide biosynthesis genes including *gna*, a putative TGC resistance gene. There were no other large deletions in T2_H3.

*Additional genes identified by genomic analysis of endpoint strains*

As described in the results section, two criteria (*p* < 0.001 in the Fisher Exact Test or the presence of identical nsSNPs in both trials) were used to create a list of 17 candidate TGC resistance genes (Table 2). A majority of the 17 genes cluster into three general groups: transcription and translation machinery, outer membrane permeability, and efflux pumps and their regulators. From the candidate genes, five genes were analyzed further due to their *p* values in both the clonal isolates and the metagenomic populations or they contained two or more identical nsSNPs in both bioreactor trials. The remaining 12 genes may also play a role in TGC resistance, and therefore, we discuss them below.

1) Transcription and translation machinery: We identified six genes involved in translation machinery and RNA processing that appear to be good candidates for reducing *A. baumannii* susceptibility to TGC. In the *rpsJ* gene encoding the S10 ribosomal protein, three mutations were detected in a loop region of the protein (Fig. 7). The ribosome recycling factor (*rrf*) contained 9 mutations including two mutations in the first 3 codons of the gene (Fig. 7). Two ribonucleases, RNase P protein (*rnpA*) and RppH (*rppH*), harbored 3 and 9 mutations, respectively. Three of the nine mutations in *rppH* truncated the resulting protein. A gene encoding one of the three elongating tRNA-Met genes contained a SNP in both trials at position 13 in the tRNA. Lastly, the RNA polymerase sigma factor D acquired 8 mutations, but the mutations were only found in one endpoint population and 25% of the isolates.

2) Outer membrane permeability: We identified mutations in outer membrane structural proteins and enzymes involved in the construction of surface polysaccharide. Outer membrane protein A (*ompA*) was mutated twice, including one nsSNP that was identical in both trials. Two genes were clustered in the K Locus, a region containing genes involved in biosynthesis of surface polysaccharides. Tyrosine-protein kinase (*wzc*) and UDP-*N*-acetyl glucosamine dehydrogenase (*gna,* Fig. 7) were present in a 22-gene region over 25 kb [49]. The region contained a 19.6 kb deletion in one T2 isolate and 41 mutations in the combined mutation dataset suggesting that it was under positive selection (Fisher Exact test, *p* < 0.001). The genes were identical to KL7 from *A. baumannii* TCDC-AB0715 and may produce legionaminic acid [49, 50].

3) Efflux pumps and their regulators: Activation of efflux systems to export toxic compounds is another mechanism for antibiotic resistance [12]. A lipid-A export permease (*msbA*) was one of the most highly mutated genes with 14 mutations which were focused in the first half of the gene [40, 41] (Fig. 7). The MATE family transporter *abeM* was mutated 4 times, including a mutation in 18 clonal isolates from both trials converting the GTG start codon to an ATG. As mentioned previously, *adeS* was mutated in every clonal isolate and contained the most mutations out of any gene. *In toto*, we identified 16 different mutations to *adeS* across our populations: 14 nsSNPs, a 21 bp deletion, and an insertion of IS Aba-1 [65] (Fig. 7).

Six additional genes passed our inclusion criteria, but either their functions were difficult to predict or their apparent role in TGC resistance was unclear. All 9 mutations in *pcaF*, a gene encoding the final step in the β-ketoadipate degradation pathway, were synonymous mutations and occurred in two T2 clonal samples (T2_E9 and T2_H9). We discovered one nsSNP and 7 indels in *ureC*, the urease alpha subunit, in 15 clonal samples and all 7 populations. Three additional genes encoded hypothetical proteins. Hypothetical proteins WM39_05440, WM39_12540, and WM39_12950 had 3, 12, and 4 mutations, respectively. There were 10 sSNPs in WM39_12540. The four mutations in WM39_12950 were in the first 75 nucleotides of the 1.4 kb gene which encodes an Hfq-like RNA binding domain.

*Mutations in* ade *family of efflux pump genes*

Interestingly, no other gene from *ade* family passed our filters even though 12 of the 18 genes contained at least one mutation (S6 Table). RND pumps consist of three proteins: membrane fusion protein (MFP: *adeA, adeF,* and *adeI*), inner membrane protein (IMP: *adeB, adeG,* and *adeJ*), and an outer membrane factor (OMF: *adeC, adeH,* and *adeK*) [12]. Due to the role these genes play in multi-drug resistance, the mutations found in the genes and their regulators are discussed below.

The *adeABC* operon is regulated by the two-component system AdeRS [14]. As described in the main text, *adeS* was highly mutated and was the most influential protein in regard to TGC resistance. The cognate response regulator, *adeR*, contained a mutation that arose to 34.3% frequency on Day 11 of Trial 1 but the mutation was not detected after Day 13. In addition, *adeB* and *adeC* were mutated four and one times, respectively. There are five genes annotated as *adeT* in the AB210 and ATCC17978 genomes [66]. The AdeT group of proteins displays similarity to MFPs of the RND pump. One of the five *adeT* genes annotated in the genome is likely co-transcribed with *adeRS* and contained a sSNP and 1-bp deletion in two clonal isolates.

The AdeIJK and AdeFGH pumps are lesser players in antibiotic resistance [12,16]. The regulator of the *adeIJK* RND pump *adeN* was mutated four times: one nsSNP, two frameshift mutations, and a nonsense mutation. In addition, *adeI* was mutated one time, and *adeJ* contained three mutations. The regulator *adeL* and the *adeFGH* operon were each mutated. *adeL* picked up three mutations and a two-nucleotide deletion less than 10 bases from its start codon. The efflux pump genes *adeF* and *adeG* contained one mutation each, whereas *adeH* was mutated three times.

These *ade* genes contained many different mutations; however, only *adeS* was reproducibly mutated in both trials and appeared to be the most prominent. The pumps may have decreased the susceptibility of *A. baumannii* to TGC; however, they were not mutated in both trials or enough times to pass the statistical test.

*Mutations in other putative TGC resistance genes*

Recent discoveries have implicated four additional genes in TGC resistance: *plsC* (WM39_ 05150), *trm* (WM39_16675), and *baeRS* (WM39_16795 and WM39_16800) [44,67,68]. No mutations were detected in *plsC* or *baeR* in either bioreactor trial. One nsSNP was observed in *baeS* (T514A) in one clonal isolate from Trial 2. No mutations were detected in *trm*; however, the gene was already truncated in the AB210M genome. When *trm* from *A. baumannii* strain ATCC 19606 was compared to the AB210M genome, T240 was deleted causing a frameshift and truncation of the protein. In addition, 37 SNPs were found in the 1.2 kb gene. The lack of mutations in these genes does not mean that they do not play a role in TGC resistance, but it highlights the plasticity of the *A. baumannii* genome.

**S1 Text References**

63. Darling AC, Mau B, Blattner FR, Perna NT. Mauve: multiple alignment of conserved genomic sequence with rearrangements. Genome Res. 2004;14: 1394–1403.

64. Clemmer KM, Bonomo RA, Rather PN. Genetic analysis of surface motility in Acinetobacter baumannii. Microbiology. 2011;157: 2534–2544. doi:10.1099/mic.0.049791-0

65. Corvec S. AmpC cephalosporinase hyperproduction in Acinetobacter baumannii clinical strains. J Antimicrob Chemother. 2003;52: 629–635. doi:10.1093/jac/dkg407

66. Bharathi Srinivasan V, Rajamohan G, Pancholi P, Marcon M, Gebreyes WA. Molecular cloning and functional characterization of two novel membrane fusion proteins in conferring antimicrobial resistance in Acinetobacter baumannii. J Antimicrob Chemother. 2011;66: 499–504. doi:10.1093/jac/dkq469

67. Li X, Liu L, Ji J, Chen Q, Hua X, Jiang Y, et al. Tigecycline resistance in Acinetobacter baumannii mediated by frameshift mutation in plsC, encoding 1-acyl-sn-glycerol-3-phosphate acyltransferase. Eur J Clin Microbiol Infect Dis Off Publ Eur Soc Clin Microbiol. 2015;34: 625–631. doi:10.1007/s10096-014-2272-y

68. Lin M-F, Lin Y-Y, Yeh H-W, Lan C-Y. Role of the BaeSR two-component system in the regulation of Acinetobacter baumannii adeAB genes and its correlation with tigecycline susceptibility. BMC Microbiol. 2014;14: 119. doi:10.1186/1471-2180-14-119
